# Efficacy of autologous stem cell at different doses combined with core decompression in the treatment of osteonecrosis of the femoral head: a systematic review and network meta-analysis

**DOI:** 10.3389/fendo.2026.1720437

**Published:** 2026-04-22

**Authors:** Mingjie Wei, Libo Yuan, Guocheng Feng, Baochuang Qi, Xiangwen Shi, Yongqing Xu

**Affiliations:** 1 Kunming Medical University, Kunming, Yunnan, China; 2 Department of Orthopedic Surgery, 920th Hospital of Joint Logistics Support Force of PLA, Kunming, Yunnan, China

**Keywords:** core decompression, doses, network meta-analysis, osteonecrosis of the femoral head, stem cells

## Abstract

**Objective:**

To compare autologous stem cell (SC) at different doses combined with core decompression (CD) versus CD alone for osteonecrosis of the femoral head (ONFH), using conversion to total hip arthroplasty (THA) as the primary outcome.

**Methods:**

PubMed, Embase, Cochrane Library, Web of Science, and CNKI were systematically searched (from inception to July 2025) to identify studies comparing different SC dosages (low dose: <1×10^7; medium dose: 1×10^7-10×10^8; high dose: >1×10^8). Conventional meta-analyses were performed using Review Manager 5.3, while network meta-analyses (NMA) were conducted with Stata 16.0. Treatment efficacy was ranked using SUCRA curves. Sensitivity analyses and funnel plots were applied to assess the robustness of the findings and potential publication bias.

**Results:**

Eighteen studies involving 1,192 patients were included. Compared with CD alone, evidence from conventional meta-analysis and network meta-analysis suggested that high-dose autologous stem cell therapy (>1×10^8 cells) combined with CD was associated with a lower risk of hip failure (conversion to THA) (OR = 0.24, 95% CI: 0.12 to 0.44). The high-dose group was also associated with a lower rate of femoral head collapse (OR = 0.24, 95% CI: 0.08 to 0.74) and lower VAS score (SMD = -1.93, 95% CI: -3.64 to -0.23). However, no statistically significant advantage of the high-dose group over the low- or medium-dose groups was observed, and no clear differences in incidence of adverse events (AEs) were detected across dose categories.

**Conclusions:**

Preliminary evidence suggests that, compared with CD alone, high-dose autologous stem cell therapy (>1×10^8 cells) combined with CD is associated with a lower risk of hip failure and a lower femoral head collapse rate, with additional improvements in pain in some comparisons. However, the certainty of evidence is limited by heterogeneity in study design, follow-up, and cell dose reporting. Future studies should emphasize standardized cell processing and intervention dosing to validate the dose–response relationship and establish the optimal clinical dosage.

**Systematic Review Registration:**

https://www.crd.york.ac.uk/prospero/display_record, identfier CRD420251154025.

## Introduction

Osteonecrosis of the femoral head (ONFH) is a disabling disease characterized by ischemic necrosis of the bone marrow and bone tissue, most commonly occurring in adults aged 30 to 50 years, with a higher incidence in males than in females (1, 2). In the United States, approximately 10,000 to 20,000 new cases are diagnosed each year (3). Although its pathogenesis has not been fully elucidated, it is known to be associated with multiple risk factors, including long-term or excessive corticosteroid use, alcohol abuse, hip trauma, and certain autoimmune diseases (4–6). Disease progression may result in subchondral fracture and collapse of the femoral head, with the majority of patients ultimately requiring total hip arthroplasty (THA) (7, 8). Therefore, early-stage treatment (ARCO stage I and II) focuses on relieving pain, improving function, and delaying disease progression, with the goal of postponing THA as long as possible (9).

Currently, the treatment of osteonecrosis of the femoral head prior to collapse mainly includes core decompression (CD), bone grafting with or without vascularization, and non-surgical strategies such as pharmacological and biophysical therapies (10, 11). As the most commonly used approach, CD alleviates pain by reducing intraosseous pressure and offers the advantage of being minimally invasive (12). However, its efficacy remains inconsistent, with short-term clinical failure rates in early-stage patients still reaching as high as 20%–70% (13), indicating that a considerable proportion of patients fail to achieve the expected therapeutic outcomes. Although bone grafting can provide subchondral structural support, promote repair, and delay femoral head collapse, it is associated with significant surgical trauma and potential complications such as donor site morbidity and nerve injury (14). In recent years, therapeutic strategies for osteonecrosis of the femoral head have gradually expanded into the field of cell-based therapies. Studies have shown that bone marrow within necrotic regions lack sufficient osteoprogenitor cells, a deficiency closely related to disease pathogenesis (15, 16). Therefore, transplanting cells with osteogenic potential, such as mesenchymal stem cells (MSCs) or bone marrow aspirate concentrate (BMAC), into necrotic regions may facilitate bone regeneration. Meanwhile, the combination of CD with SC therapy, particularly autologous SC transplantation, has attracted widespread attention. Preliminary clinical results suggest its potential in alleviating pain, reducing lesion size, and delaying joint replacement (17).

Several previous meta-analyses have summarized clinical evidence regarding the efficacy of autologous SCs combined with CD in the treatment of ONFH, consistently confirming that the combined therapy is more effective in slowing disease progression (18–20). A recent meta-analysis further demonstrated that both MSC and BMAC therapies can effectively prevent ONFH progression and reduce the conversion rate to THA (21). However, all prior meta-analyses have focused solely on the clinical efficacy of SC combined with CD or single SC injection in ONFH patients, while neglecting the potential influence of SC dosage on therapeutic outcomes. Therefore, it is unclear whether the benefit is consistent across the wide range of cell doses used in clinical practice. Hernigou et al. (22, 23) reported the existence of a theoretical cellular threshold for ONFH repair (approximately 24,000 to 35,000 MSCs), with clinical prognosis being significantly associated with the number of progenitor cells implanted. Nevertheless, current clinical studies exhibit substantial heterogeneity in cell preparation, counting methodologies, and final injection dosages, which may account for variations in efficacy and hinder the standardization of treatment protocols (24). Therefore, simply comparing combined therapy with decompression alone is no longer sufficient to guide clinical decision-making. Whether a higher dose of autologous SC confers superior clinical benefits, particularly in preventing the progression to THA, remains an unresolved and critical question.

This study, for the first time, performed conventional meta-analysis and network meta-analysis (NMA) to compare different autologous SC dosage ranges combined with CD versus CD alone for ONFH. The primary objective was to determine whether any SC dose category reduces the rate of hip failure, defined as conversion to THA at the latest follow-up. Secondary outcomes included femoral head collapse, pain, function, and adverse events (AEs). The aim is to provide evidence supporting the formulation of standardized stem cell therapy protocols and individualized precision treatments.

## Materials and methods

This study was conducted in accordance with the Preferred Reporting Items for Systematic Reviews and Meta-Analyses (PRISMA) guidelines for network meta-analyses (25, 26). The study protocol was prospectively registered in the PROSPERO database (CRD420251154025).

### Search strategies

The data sources searched included PubMed, Embase, Web of Science, the Cochrane Library, and the China National Knowledge Infrastructure (CNKI), covering the period from the inception of each database to July 1, 2025. A combination of subject headings and free-text terms was employed, focusing on “Stem Cells” AND “Osteonecrosis” AND “Core Decompression” and their synonyms or abbreviations. The detailed search strategies for each database are provided in the **Supplementary Material**. Additionally, to minimize potential omissions, we manually screened the reference lists of relevant reviews and included articles. No language restrictions were applied in this search.

### Eligibility criteria

This study established inclusion and exclusion criteria based on the PICOS framework: (1) Participants (P): adult patients with osteonecrosis of the femoral head confirmed by imaging, with no restrictions on etiology or disease stage. (2) Interventions (I): transplantation of autologous bone marrow–derived SCs at different doses, including SCs combined with standard treatments such as CD. SC was used as a shorthand for autologous bone marrow/peripheral blood–derived cell therapies delivered locally to the femoral head to augment core decompression. Eligible products included (i) BMAC/BMC, (ii) buffy coat–based concentrates used with grafting, and (iii) culture-expanded mesenchymal stromal cells or osteoblastic cells administered as a defined cell number. (3) Comparators (C): standard interventions (e.g., core decompression, non-surgical therapies). (4) Outcomes (O): the primary outcome was conversion to THA at the latest follow-up. Secondary outcomes included rate of femoral head collapse, hip function (Harris Hip Score and WOMAC score), pain (VAS score), and the incidence of AEs. (5) Study design (S): limited to clinical controlled trials. Exclusion criteria included: non-controlled studies, duplicate publications, studies involving allogeneic stem cells, and studies with incomplete data or insufficient data for extraction.

### Study selection

The study selection process was conducted independently by two researchers (LY, GF). First, all records retrieved from the databases were imported into Endnote 20 (Clarivate Analytics), where duplicate entries were automatically removed. Subsequently, the two researchers independently screened the titles and abstracts of the remaining studies according to the predefined inclusion and exclusion criteria, excluding obviously irrelevant articles. For studies that appeared to meet the criteria, the full texts were obtained and assessed in detail to determine final eligibility. Throughout the process, the screening results were cross-checked between the two researchers, and any disagreements were resolved by consultation with a third researcher. The final selection process and results will be presented in detail using a PRISMA flow diagram.

### Data extraction

Data extraction was independently performed by two researchers using a predesigned, standardized Excel form (XS, BQ). The extracted information primarily included: (1) study characteristics (e.g., first author, year of publication, country, study design); (2) participant characteristics (e.g., sample size, patient age, sex, etiology, ARCO stage); (3) details of the interventions, such as stem cell source, isolation method, dose, route of administration, and control interventions; and (4) outcomes, including conversion to THA, femoral head collapse, Harris Hip Score, WOMAC score, VAS score, and incidence of AEs. In cases of missing or uncertain data, the corresponding authors were contacted by email for verification. The two independently extracted datasets were cross-checked, and any discrepancies were resolved through discussion with a third researcher. The reported dose metrics varied substantially across studies, and were often unavailable at the per-hip level; therefore, a threshold distribution analysis was not feasible from published data alone. We prespecified dose categories on a log scale as follows: low dose <1×10^7 cells, medium dose 1×10^7 to 1×10^8 cells, and high dose >1×10^8 cells (27), based on the orders of magnitude of administered cell numbers reported in the included studies.

### Quality assessment and GRADE

This study employed two validated tools to assess methodological quality. For randomized controlled trials (RCTs), the Cochrane Risk of Bias (RoB) tool was used to evaluate six domains: selection bias, performance bias, detection bias, attrition bias, selective reporting, and other potential sources of bias. For retrospective cohort studies or case-control studies, the Newcastle–Ottawa Scale (NOS) was applied, assessing three dimensions: selection of study subjects, comparability between groups, and outcome assessment. The evaluation process was conducted independently by two researchers (LY, GF), with any disagreements resolved through discussion with a third researcher. Based on the assessment results, we determined the risk of bias for each individual study and considered the influence of study quality on the overall body of evidence in the integrated discussion. All evaluation results will be presented in both tabular form and summary figures.

Additionally, we evaluated the certainty of evidence for the key outcomes (conversion to THA and femoral head collapse) using an adapted GRADE framework (28, 29). Certainty ratings considered five methodological domains: risk of bias, inconsistency, indirectness, publication bias, and imprecision. For each treatment comparison, the overall certainty was summarized as high, moderate, low, or very low.

### Statistical analysis

Conventional meta-analyses were performed using Review Manager (RevMan) version 5.3 to pool direct comparative evidence. For dichotomous variables, odds ratios (ORs) with 95% confidence intervals (CIs) were calculated as effect measures. For continuous variables, standardized mean differences (SMDs) with 95% CIs were used. Subgroup analyses were conducted based on different stem cell dosages (low, medium, and high) for each outcome measure. Heterogeneity was assessed using the *I*² statistic, with *I*² > 50% indicating substantial heterogeneity, in which case a random-effects model was applied; when *I*² ≤ 50%, a fixed-effects model was used. A *P*-value of <0.05 was considered statistically significant.

NMA was conducted in Stata/SE 16.0 using the “mvmeta” and “network” packages. A network plot was generated to visualize the comparative relationships among different intervention dosages, where node size represented sample size and line thickness indicated the number of direct comparison studies. Global inconsistency tests were performed to evaluate the consistency assumption; when the Z-test yielded *P* > 0.05, a consistency model was applied. In addition, the surface under the cumulative ranking curve (SUCRA) was used to rank the efficacy of each intervention. Publication bias was assessed using funnel plots, and sensitivity analyses were performed to test the robustness of the results.

## Results

### Search results and study characteristics

**Figure 1:**
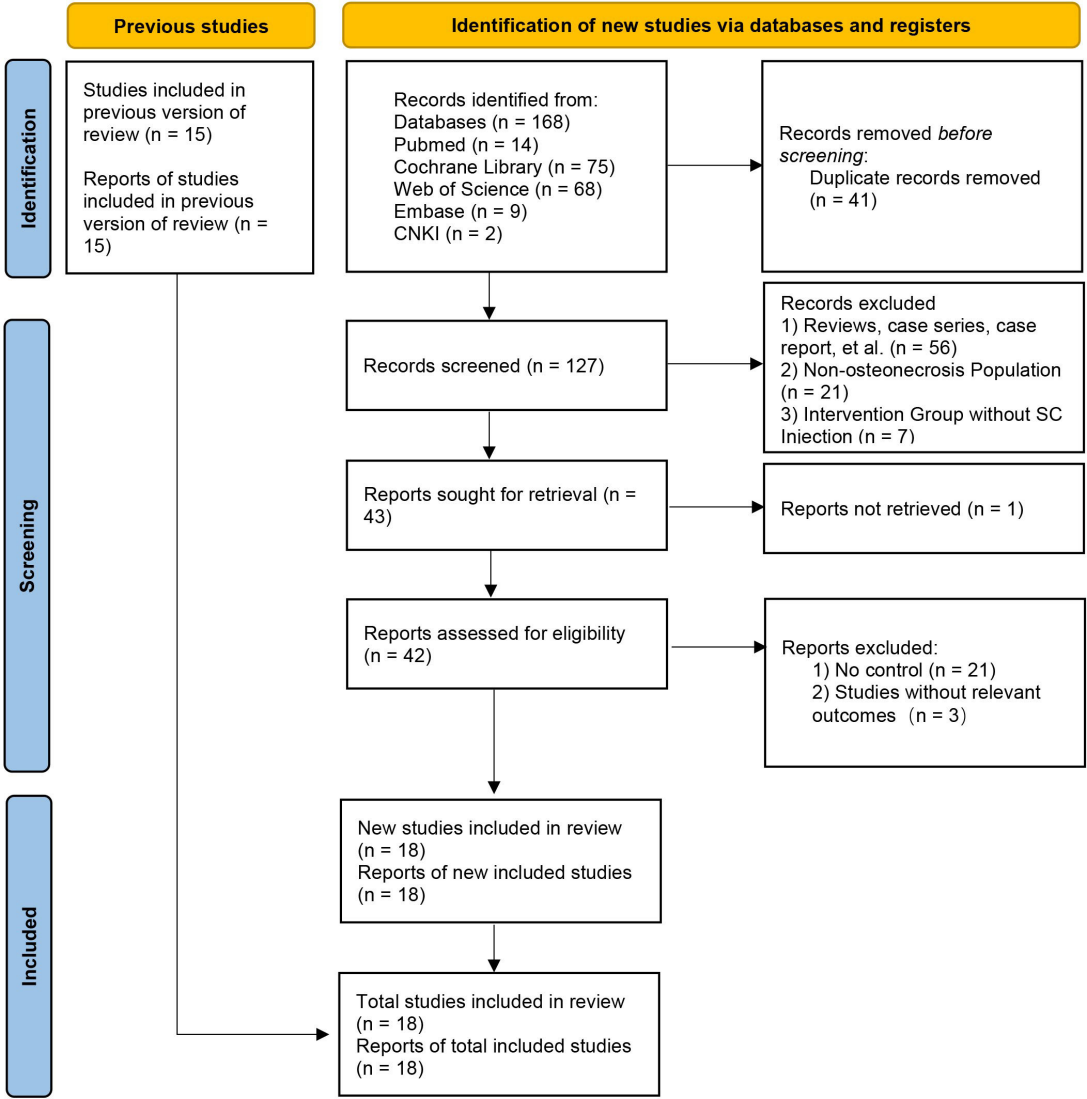
PRISMA flow diagram of the study selection process.

A total of 168 relevant records were initially identified through the preliminary search. After further rigorous screening, 18 clinical trials (30–47) were ultimately included in the meta-analysis, comprising 17 studies in English and 1 study in Chinese. The detailed search and selection process is illustrated in **Figure 1**.

Among the 18 included studies, 14 were randomized controlled trials (RCTs), 2 were prospective non-randomized controlled trials, and 2 were retrospective cohort or case-control studies. Seven studies were conducted in China, four in Belgium, two in India, and the remaining five were carried out in Germany, France, the United States, South Korea, and Iran, respectively. In total, the 18 studies enrolled 1,192 patients with ONFH. Among these, 12 studies reported 212 cases with alcohol-induced ONFH, 13 studies reported 321 cases with steroid-induced ONFH, and 12 studies included 202 patients with idiopathic ONFH. Patient characteristics are summarized in **Table 1**. Regarding SC characteristics, most cell products were autologous and bone marrow–derived; one trial used autologous G-CSF-mobilized peripheral blood stem cells delivered by targeted intra-arterial infusion (42). Dose metrics included total nucleated cells (TNC), mononuclear cells (MNC), CFU-F–based progenitor estimates, and culture-expanded cell concentrations; in 6 studies, quantitative cell counts were not reported. The injected cell dose ranged from 9.0 × 10^4 to 3 × 10^9 cells. Modes of administration included direct injection into the necrotic region (N = 16), anchoring onto the bone surface (N = 1), and targeted intra-arterial infusion (N = 1). Follow-up duration ranged from 2 to 25 years, with two studies reporting mean follow-up periods exceeding 10 years. Detailed SC characteristics are provided in **Table 2**.

**Table 1:** Main characteristics of patients in studies included.

Author	Year	Country	Study design	Age (SC vs control, Years ± SD, range)	BMI (kg/m^2^) (SC vs control)	Number of hips (SC vs control)	Etiologic factors (SC vs control)			ARCO stage (SC vs control)			Follow-up (year)
							Alcohol	Steroids	Idiopathic	I	II	III and IV	
Chang et al (30)	2010	China	RCT	33.55±8.36	NR	8 vs 8	NR	NR	NR	NR	7 vs 7	1 vs 1	2
Gangji et al (31)	2011	Belgium	Prospective non-RCT	42.2±2.6 vs 45.7 ± 2.8	NR	13 vs 11	1 vs 1	11 vs 9	1 vs 1	2 vs 2	11 vs 9	NR	5
Gangji et al (32)	2016	Belgium	RCT	50.8 ± 13.2 vs 51.1 ± 10.6	NR	30 vs 30	NR	NR	NR	NA	NR	NR	3
Hauzeur et al (33)	2018	Belgium	RCT	48.0 ± 2.8 vs 49.7 ± 3.2	25.35 ± 0.75 vs 24.53 ± 0.96	23 vs 23	8 vs 7	12 vs 13	1 vs 3	2 vs 4	10 vs 9	11 vs 10	2
Hauzeur et al (34)	2019	Belgium	RCT	50 ± 12 vs 51 ± 10	27 ± 5 vs 26 ± 5	26 vs 27	11 vs 7	19 vs 16	1 vs 3	10 vs 10	16 vs 17	NR	3
Hernigou et al (35)	2018	France	Prospective non-RCT	36 ± 6.98	NR	125 vs 125	NR	NR	NR	NR	NR	NR	25
Hoogervorst et al (36)	2022	USA	Retrospective cohort	33.1 ± 10.0 vs 39.8 ± 12.6	28.3 ± 6.5 vs 29.2 ± 8.7	61 vs 24	9 vs 2	50 vs 20	2 vs 2	6 vs 6	45 vs 16	10 vs 2	5
Jayankura et al (37)	2023	China	RCT	46 ± 10 vs 45 ± 10	NR	25 vs 29	13 vs 24	6 vs 7	NR	5 vs 3	19 vs 24	1 vs 2	2
Kang et al (38)	2018	South Korea	Case-control studies	46.0 ± 9.3 vs 47.3 ± 9.7	NR	53 vs 53	19 vs 19	5 vs 5	24 vs 24	1 vs 1	29 vs 29	23 vs 23	4.53
Li et al (39)	2020	China	RCT	34.1 ± 8.0 vs 38.2 ± 8.1	NR	21 vs 20	6 vs 5	10 vs 9	5 vs 6	10 vs 9	11 vs 11	NR	10
Li et al (40)	2021	China	RCT	35.4 ± 11.1 vs 39.4 ± 10.4	NR	22 vs 29	5 vs 9	8 vs 3	9 vs 17	1 vs 1	19 vs 20	2 vs 8	2
Ma et al (41)	2014	China	RCT	35.60 ± 8.05 VS 34.78 ± 11.48	NR	25 vs 24	4 vs 3	13 vs 13	6 vs 6	3 vs 4	17 vs 15	5 vs 5	2
Mao et al (42)	2015	China	RCT	34.60 ± 11.50 vs 36.12 ± 11.34	NR	48 vs 41	18 vs 14	16 vs 15	14 vs 12	8 vs 10	29 vs 23	11 vs 8	3
Pepke et al (43)	2016	Germany	RCT	44.3 ± 3.4 vs 44.5 ± 3.3	NR	11 vs 14	NR	NR	NR	NR	NR	NR	2
Rastogi et al (44)	2013	India	RCT	34.67 ± 7.02 vs 33.0 ± 7.71	NR	30 vs 30	2 vs 6	10 vs 8	14 vs 12	7 vs 7	11 vs 11	12 vs 12	2
Sen et al (45)	2012	India	RCT	66.19 ± 13.04 vs 65.72 ± 15.24	NR	26 vs 25	NR	NR	NR	6vs 7	9 vs 4	11 vs 8	2
Tabatabaee et al (46)	2015	Iran	RCT	31 ± 11.4 vs 26.8 ± 5.8	NR	14 vs 14	NR	10 vs 9	4 vs 5	3 vs 2	9 vs 7	2 vs 5	2
Zhao et al (47)	2012	China	RCT	32.7 ± 10.5 vs 33.8 ± 7.70	NR	53 vs 51	11 vs 8	11 vs 13	17 vs 13	3 vs 2	50 vs 49	NR	5

ARCO, Association Research Circulation Osseous; BMI, Body mass index; NR, Not reported; SC, Stem cell. SD, Standard deviation.

**Table 2:** Preparation and intervention characteristics of autologous bone marrow/peripheral blood-derived SC.

Author	Year	Source	Cell product type	Volume of whole	Isolation	Preparation	Dose metric used	Dose/Volume administered	Route	Control
Chang et al (30)	2010	Autologous BM	BMSC + DBM	15-25 mL	Centrifugation	Culture-expanded BMSCs + DBM	Expanded cells concentration	2 × 10^6^/NR	Instilled into the necrotic zone	CD
Gangji et al (31)	2011	Autologous BM	BMC	NR	Centrifugation	NR	MNC; CD34%; CFU-F	92.6 × 10^7^/49.7±2.3 mL	Injection into the necrotic zone	CD
Gangji et al (32)	2016	Autologous BM	BMC	410.6 ± 84.9 mL	Centrifugation	NR	NR	20 × 10^6^/41.8±10.9 mL	Instilled into the necrotic zone	CD
Hauzeur et al (33)	2018	Autologous BM	BMAC + CD	400 mL	Centrifugation	NR	TNC; CD34%; CFU-F	19.45 × 10^6^/48.33±1.16 mL	Injection into the necrotic zone	CD + saline injection
Hauzeur et al (34)	2019	Autologous BM	BMAC	400 ± 85 mL	Centrifugation	NR	TNC; CD34%; CFU-F	9.2 × 10^6^/40±11 mL	Injection into the necrotic zone	CD + osteoblastic cell
Hernigou et al (35)	2018	Autologous BM	BMSC + CD	152 ± 16 mL	Centrifugation	NR	CFU-F	9.0 × 10^4^/20 mL	Injection into the necrotic zone	CD
Hoogervorst et al (36)	2022	Autologous BM	BMAC + CD	60 mL	Centrifugation	NR	NR	1.81 × 10^9^/NR	Injection into the necrotic zone	CD
Jayankura et al (37)	2023	Autologous BM	Osteoblastic cells + CD	30-50 mL	Centrifugation	NR	Expanded cells	20 × 10^6^/5 mL	Injection into the necrotic zone	CD + saline injection
Kang et al (38)	2018	Autologous BM	BMSC + CD	100-120 mL	Centrifugation	NR	CFU-F	13.97 × 10^6^/15 mL	Instilled into the necrotic zone	CD
Li et al (39)	2020	Autologous BM	BBC + CD	NR	Centrifugation	NR	TNC	3 × 10^9^/NR	Instilled into the necrotic zone	CD + autologous BG
Li et al (40)	2021	Autologous BM	ACD + BBC + ABR	30-50 mL	Centrifugation	NR	NR	3 × 10^9^/3 mL	Injection into the necrotic zone	ACD + β-TCP + ABR grafting
Ma et al (41)	2014	Autologous BM	BBC + CD	NR	Centrifugation	Bone marrow buffy coat seeded on the cylindrical bone before implantation	TNC	3 × 10^9^/NR	Anchor on the bone surface	CD + autologous BG
Mao et al (42)	2015	Autologous peripheral blood	PBSC	60 ± 15.74 mL	Centrifugation	G-CSF at a dosage of 10 μg/kg for 4 days	CD34%	2.47 × 10^8^/NR	Targeted intra-arterial infusion of PBSCs	Porous tantalum rod implantation
Pepke et al (43)	2016	Autologous BM	BMAC + CD	200-220 mL	Centrifugation	NR	NR	2 × 10^6^/10 mL	Instilled into the necrotic zone	CD
Rastogi et al (44)	2013	Autologous BM	Bone marrow-derived mononuclear cells + CD	60-70 mL	Centrifugation	NR	NR	1.1 × 10^8^/5 mL	Injection into the necrotic zone	CD + unprocessed bone marrow
Sen et al (45)	2012	Autologous BM	Bone marrow-derived mononuclear cells + CD	120-180 mL	Centrifugation	NR	MNC; CD34%	5 × 10^8^/2 mL	Injection into the necrotic zone	CD
Tabatabaee et al (46)	2015	Autologous BM	BMSC + CD	200 mL	Centrifugation	NR	MNC	5 × 10^8^/NR	Instilled into the necrotic zone	CD
Zhao et al (47)	2012	Autologous BM	BMSC + CD	NR	Centrifugation	NR	NR	2 × 10^6^/2 mL	Injection into the necrotic zone	CD

ABR, Angioconductive bioceramic rod; BBC, Bone marrow buffy coat; BG, Bone graft; BMAC, Bone marrow aspirate concentrate; BMC, Bone marrow concentrate; BMSC, Bone marrow mesenchymal stem cell; CD, Core decompression; CFU-F, Colony forming units-fibroblast; DBM, Decalcified bone matrix; MNC, Mononuclear cells; NR, Not reported; PBSC, Peripheral blood stem cell; SC, Stem cell. TNC, Total nucleated cell.

### Risk of bias assessment

**Figure 2:**
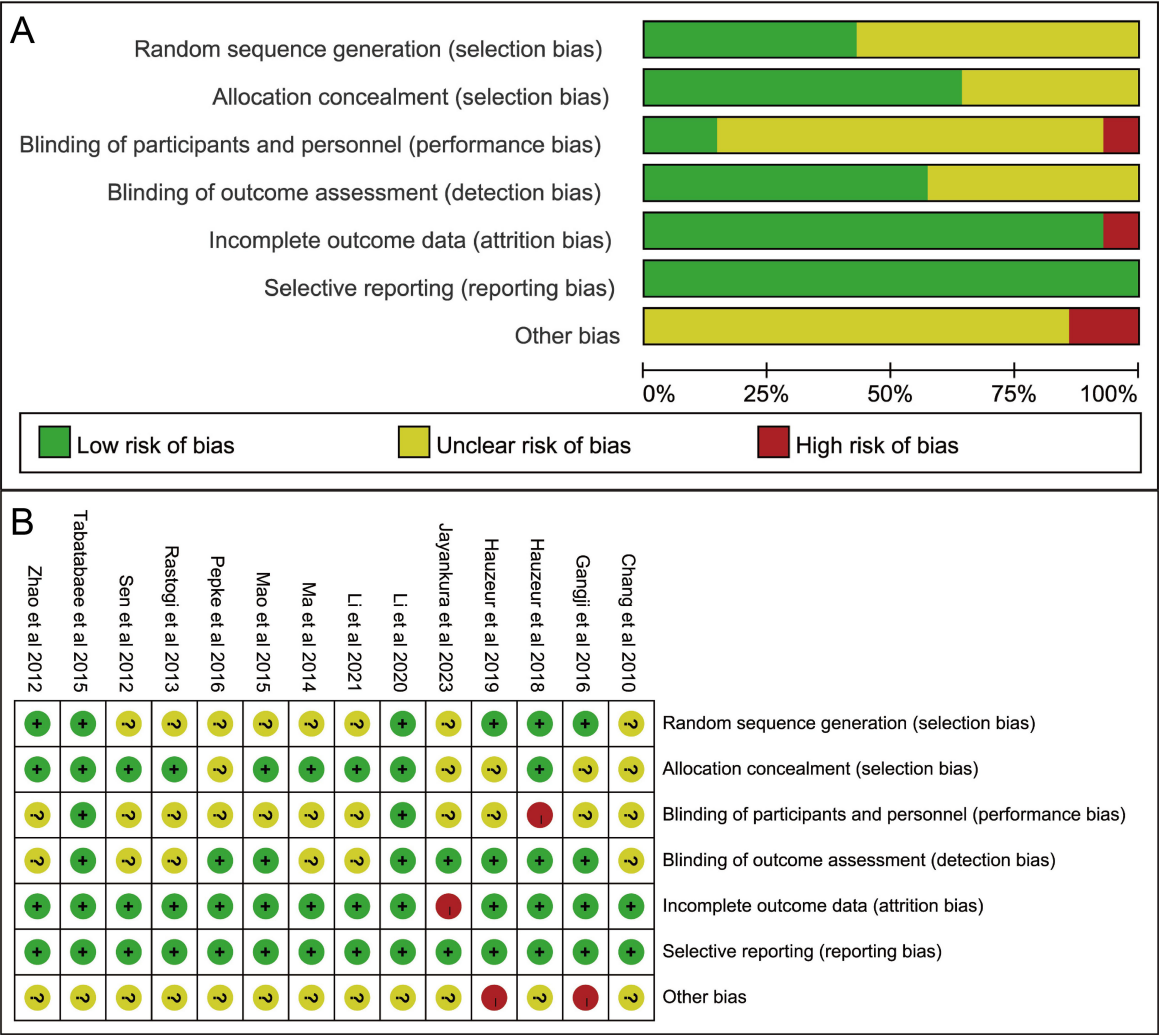
The risk of bias graph of included studies. **(A)** Risk of bias graph; **(B)** Risk of bias summary.

For the RCTs, most studies were evaluated to have either low or unclear risk of bias (**Figures 2A, B**). Specifically, none of the studies were rated as having a low risk of bias across all domains. Four studies had a high risk of bias in one domain: two due to potential funding from companies involved in SC preparation, one because of a high dropout rate, and one for explicitly lacking blinding of clinicians. Six studies reported detailed methods of random sequence generation, nine provided information on allocation concealment, and eight clearly stated that outcome assessment was blinded. All studies were considered to have a low risk of bias in terms of selective reporting. The results of the risk of bias assessment for RCTs are presented in **Figures 2A, B**. For the four controlled observational studies, the Newcastle–Ottawa Scale (NOS) was applied. Two studies scored 7–9, indicating high quality, while two studies scored 5–6, indicating moderate quality. The results of NOS-based quality assessment are provided in **Supplementary Table 1**.

### Results of conventional meta-analysis

#### Hip failure (conversion to THA)

**Figure 3:**
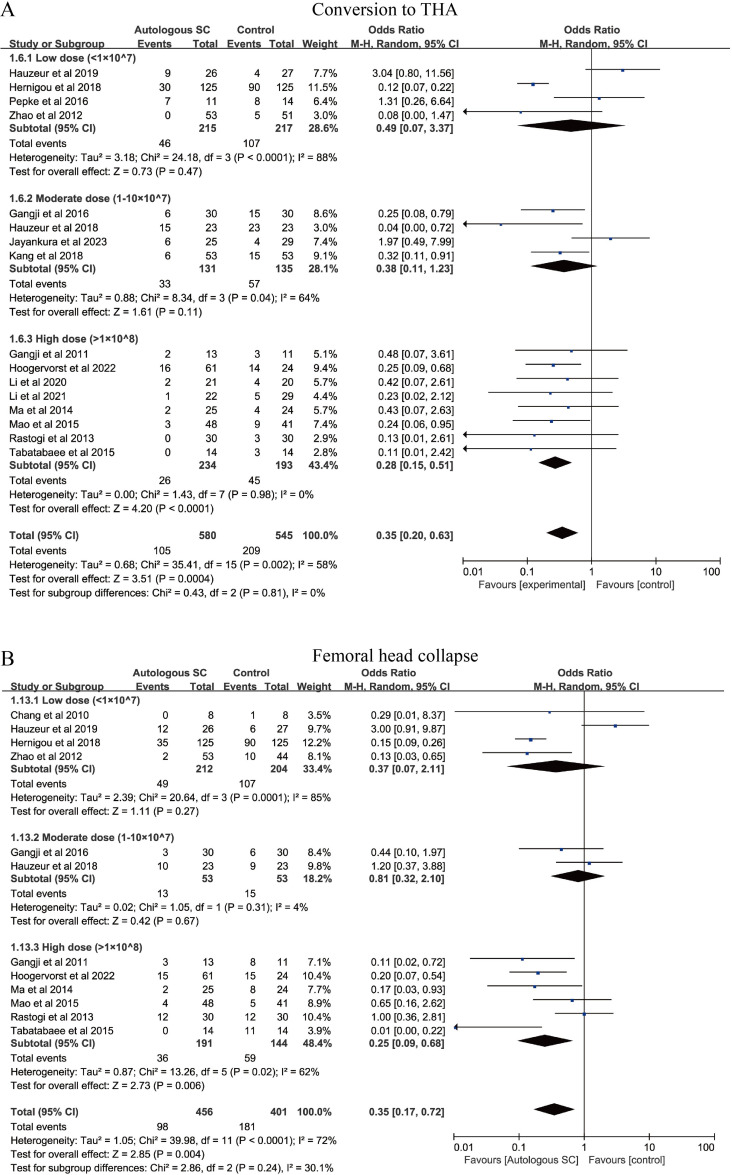
Forest plot illustrating the effects of different SC dosages on rates of conversion to THA and femoral head collapse. **(A)** conversion to THA. **(B)** femoral head collapse.

We evaluated hip failure, defined as conversion to THA, after SC combined with CD (**Figure 3A**). 16 studies (1125 patients) reported THA conversion, involving the low-, medium- and high-dose subgroups. Compared with the control group, the high-dose SC subgroup was associated with a significantly lower risk of hip failure (OR = 0.28, 95% CI: 0.15–0.51, *P* < 0.0001, *I*² = 0%). In contrast, the low- and medium-dose subgroups did not show statistically significant reduction in hip failure compared with CD alone. Subgroup analysis based on different follow-up durations showed that in the ≥2 years subgroup, SC combined with CD treatment significantly reduced the risk of hip failure (**Supplementary Figure 1**). However, the follow-up duration subgroup may not be a significant factor influencing the heterogeneity of hip failure.

We additionally summarized reported survival time of hips in four studies (206 patients) (**Supplementary Figure 2**). Due to heterogeneous definitions and inconsistent reporting of time-to-event data, these results should be interpreted cautiously. The pooled estimate did not reach statistical significance (*P* = 0.05).

#### Femoral head collapse

Femoral head collapse is an important indicator of disease progression in ONFH (48). Subgroup analyses were performed to evaluate the association between SC dose categories and the risk of femoral head collapse (**Figure 3B**). Compared with CD alone, the high-dose SC subgroup was associated with a significantly lower collapse rate (OR = 0.35, 95% CI: 0.17-0.72, *P* = 0.004), with substantial heterogeneity (*I*² = 72%). No statistically significant reduction in collapse was observed in the low- or medium-dose subgroups compared with CD alone. Subgroup analysis results suggested that in the ≥2 years subgroup, SC combined with CD treatment may be associated with a lower risk of hip failure (**Supplementary Figure 3**).

Two studies additionally reported time to collapse after treatment (**Supplementary Figure 4**). Given the limited number of studies and variability in reporting of time-to-event information, this analysis should be interpreted cautiously. The pooled estimate did not reach conventional statistical significance (SMD = 0.35, 95% CI: –0.02 to 0.72, *P* = 0.06).

#### VAS score

**Figure 4:**
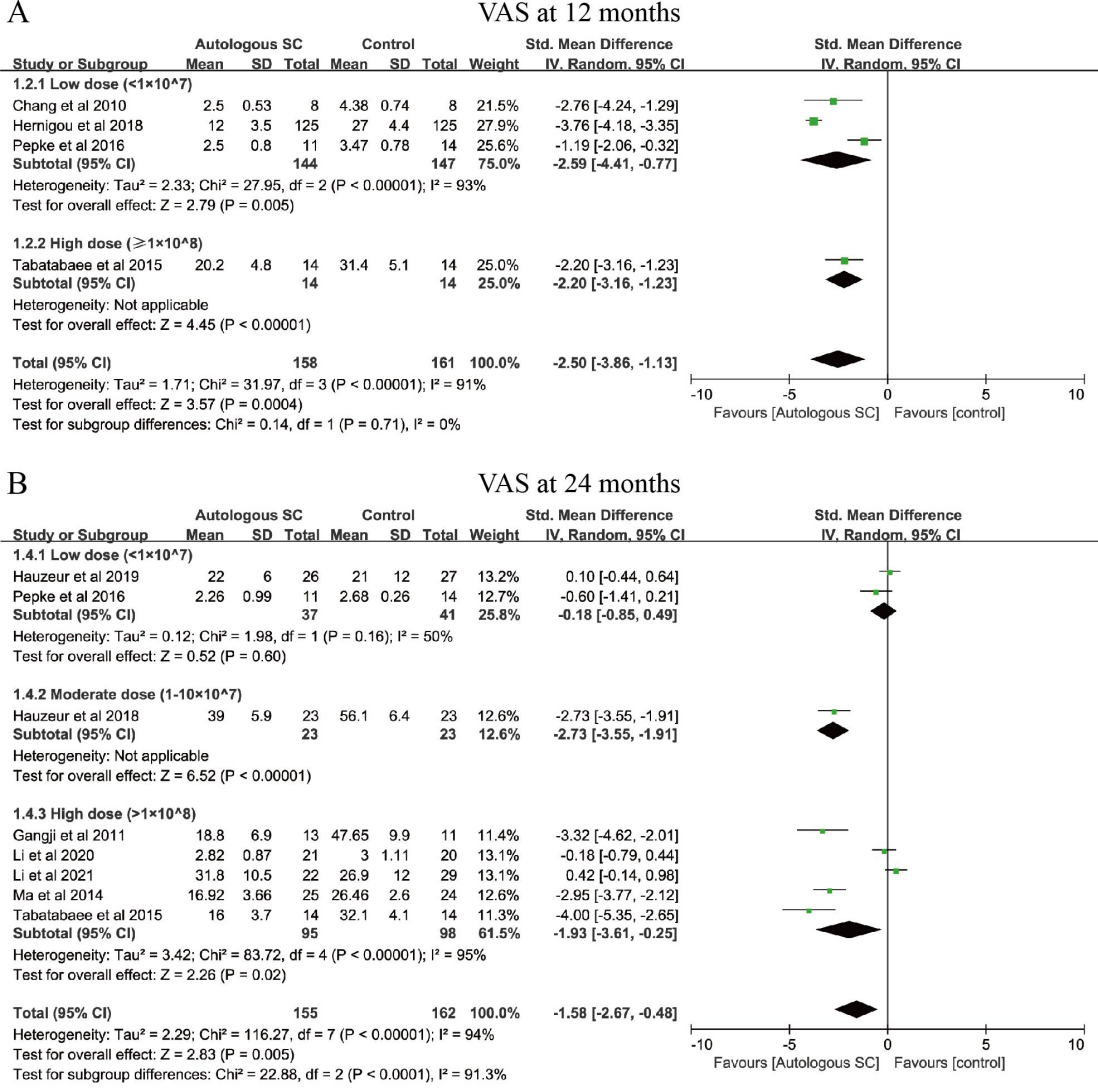
Forest plot illustrating the effects of different SC dosages on VAS score. **(A)** VAS score at 12 months. **(B)** VAS score at 24 months.

Additionally, VAS scores related to ONFH are primarily used for clinical assessment of hip joint symptoms (49). Patients were stratified by dosage into low-dose (<1×10^7), medium-dose (1×10^7-10×10^8), and high-dose (>1×10^8) groups, and subgroup analyses were performed at different follow-up time points. The 12-month VAS score analysis included four studies (319 patients), with 158 in the autologous SC group and 161 in the control group (**Figure 4A**). Compared with controls, the low-dose SC subgroup showed a significant reduction in VAS scores (SMD = -2.59, 95% CI: -4.41 to -0.77, *P* = 0.005), with notable heterogeneity (*I*² = 93%). Since only one study was included in the high-dose subgroup, further interpretation of this result was limited. The 24-month VAS score analysis also included four studies (319 patients), divided into three subgroups (**Figure 4B**). At this time point, the high-dose SC subgroup significantly reduced VAS scores compared with controls (SMD = -1.93, 95% CI: -3.61 to -0.25, *P* = 0.02), though substantial heterogeneity was again present (*I*² = 95%). The low- and medium-dose subgroups, containing two and one studies respectively, provided limited interpretability. Notably, the pain-relieving effect of SC appeared to decline over time: compared with the 12-month analysis (SMD = -2.50, 95% CI: -3.86 to -1.13, *P* = 0.0004), the effect at 24 months was attenuated (SMD = -1.58, 95% CI: -2.67 to -0.48, *P* = 0.005). This may reflect a limitation in the sustained analgesic benefit of SC with longer follow-up. However, given the limited number of studies, current evidence is insufficient to establish a potential association between high- or low-dose SC and long-term VAS score improvement.

#### Function score

**Figure 5:**
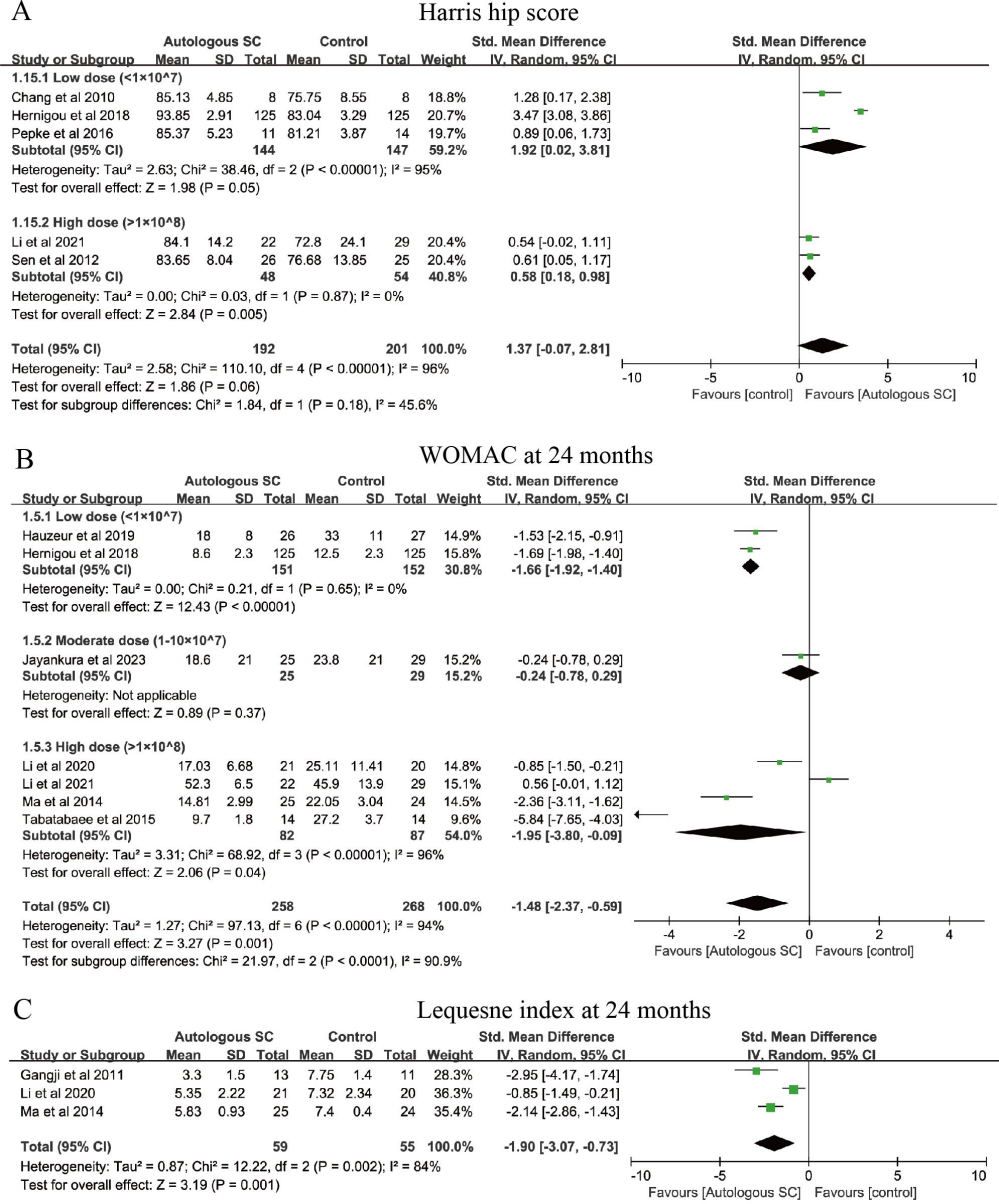
Forest plot illustrating the effects of different SC dosages on hip functions. **(A)** Harris hip score. **(B)** WOMAC score at 24 months. **(C)** Lequesne index at 24 months.

The Harris Hip Score encompasses four domains, including pain, function, deformity, and range of motion, and is commonly used to evaluate treatment efficacy in osteonecrosis of the femoral head (50). Five studies involving 393 patients reported HHS outcomes (**Figure 5A**). Compared with controls, the low-dose subgroup (SMD = 1.92, 95% CI: 0.02 to 3.81, *P* = 0.05) and the high-dose subgroup (SMD = 0.58, 95% CI: 0.18 to 0.98, *P* = 0.005) may confer improvements in HHS. However, given the small number of studies and substantial heterogeneity, these findings should be interpreted cautiously. Seven studies (526 patients) reported 24-month WOMAC scores (**Figure 5B**); relative to controls, both the low-dose (SMD = −1.66, 95% CI: −1.92 to −1.40, *P* < 0.00001) and high-dose groups (SMD = −1.95, 95% CI: −3.80 to −0.09, *P* = 0.04) showed reductions in WOMAC. Additionally, three studies reported the 24-month Lequesne index (**Figure 5C**), indicating that SC intervention may lower the index and improve hip function (SMD = −1.90, 95% CI: −3.07 to −0.73, *P* = 0.001). In light of pronounced heterogeneity (*I*² = 84%), these results should be interpreted with caution.

Given the significant heterogeneity observed in the pooled analysis of VAS and WOMAC scores at 24 months, a subgroup analysis was conducted to explore the factors contributing to the significant heterogeneity (**Supplementary Figure 5**). However, the results of the subgroup analysis indicated that follow-up duration (< 2 years and ≥ 2 years) may not be a significant factor influencing the heterogeneity.

#### ARCO stage

The ARCO classification (Association Research Circulation Osseous) is currently the most widely used and comprehensive staging system for osteonecrosis of the femoral head, consisting of four stages (51). Among these, stages I and II represent early disease, whereas stages III and IV indicate collapse. We compared changes in ARCO stages before and after SC treatment, focusing on the medium- and high-dose subgroups (**Supplementary Figure 6**). For ARCO stage I, no significant changes were observed in patient numbers after treatment with either medium- or high-dose SC compared with baseline (*P* > 0.05). Similarly, consistent with stage I findings, no significant changes were detected in the numbers of patients classified as stage II or III following medium- or high-dose SC intervention (*P* > 0.05).

#### Incidence of AEs

Ten studies (272 patients) reported the incidence of AEs, covering high-, medium-, and low-dose subgroups (**Supplementary Figure 7**). Because five studies reported zero AEs in both arms, pooled analysis was limited to the remaining five studies. Overall, events were infrequent and generally mild. Hauzeur et al. (33) reported no serious AEs; the recorded events consisted mainly of local pain (greater trochanter/iliac crest), transient fever (<24 h), and nausea. In Hauzeur et al. (34), treatment-emergent events were described as uncommon and transient, with pyrexia being the most frequently noted event. Jayankura et al. (37) found no treatment-related serious AEs; the only serious event (procedural pain) was attributed to bone marrow aspiration, and treatment-related nonserious AEs were rare. Similarly, Gangji et al. (31) reported no serious aspiration- or implantation-related reactions, with minor events limited to aspiration-site pain, local pain at the decompression site, and one positive bone marrow culture treated with antibiotics without clinical sepsis. Mao et al. (42) documented one postoperative infection in the control group and one porous tantalum rod displacement in the combination group. Compared with controls, SC intervention did not show a significant effect in reducing AE incidence (*P* = 0.33). Given the small number of analyzable studies and sparse events, these findings should be interpreted cautiously.

Through conventional meta-analysis, we found that high-dose SC treatment may play a beneficial role in reducing rate of THA conversion, incidence of hip collapse, VAS and WOMAC scores. However, due to insufficient and inconsistent follow-up data, it remains unclear whether high-dose SC can provide sustained long-term improvements for patients.

### Results of network meta-analysis

#### Hip failure and femoral head collapse

**Figure 6:**
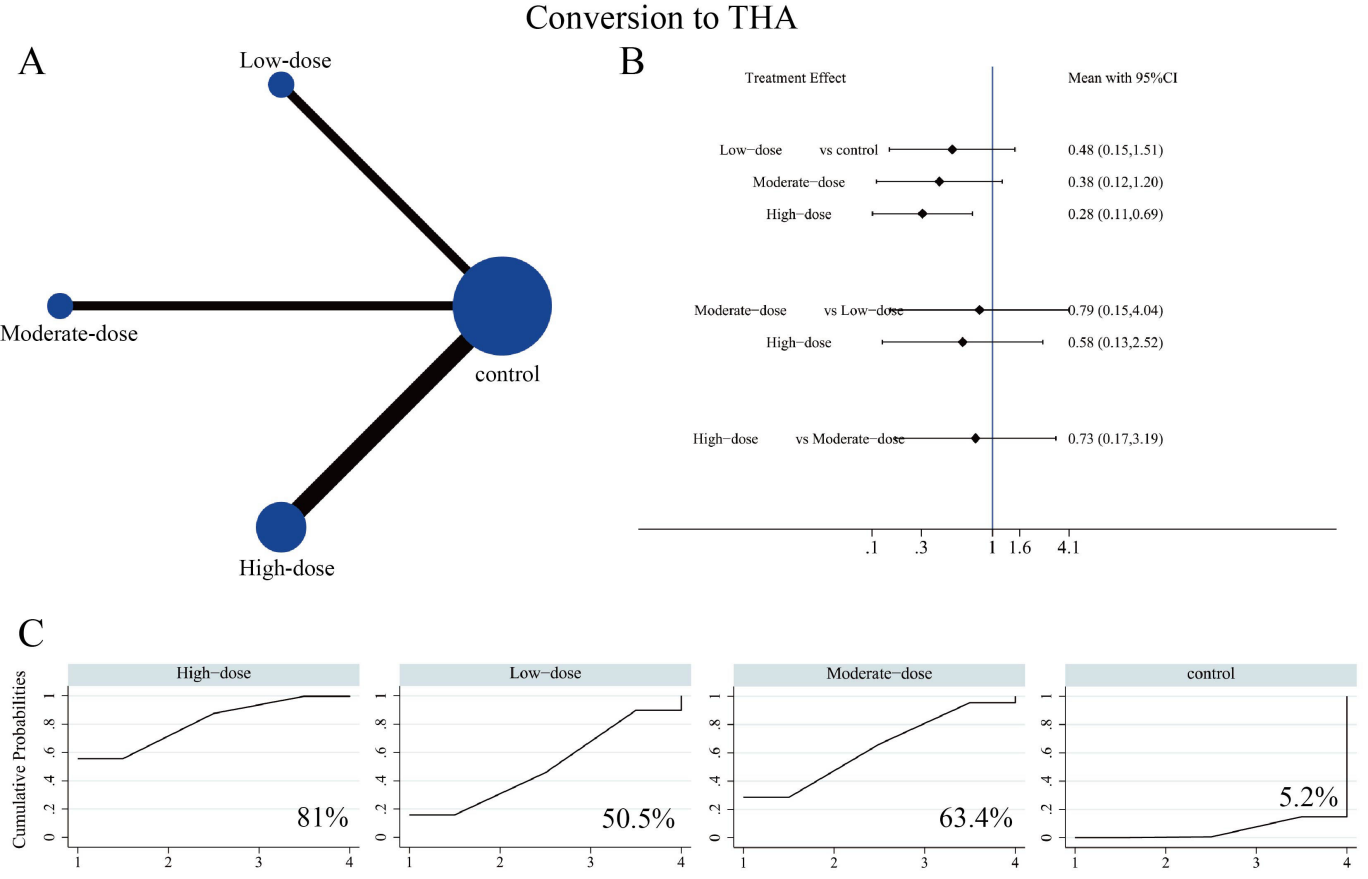
Network meta-analysis of the effects of different SC dosages on rate of conversion to THA. **(A)** Evidence network diagram for conversion to THA. **(B)** Forest plot presenting comparative evidence of different SC dosages on conversion to THA. **(C)** SUCRA curves and surface under the cumulative ranking curve (%) for conversion to THA after SC intervention.

**Figure 7:**
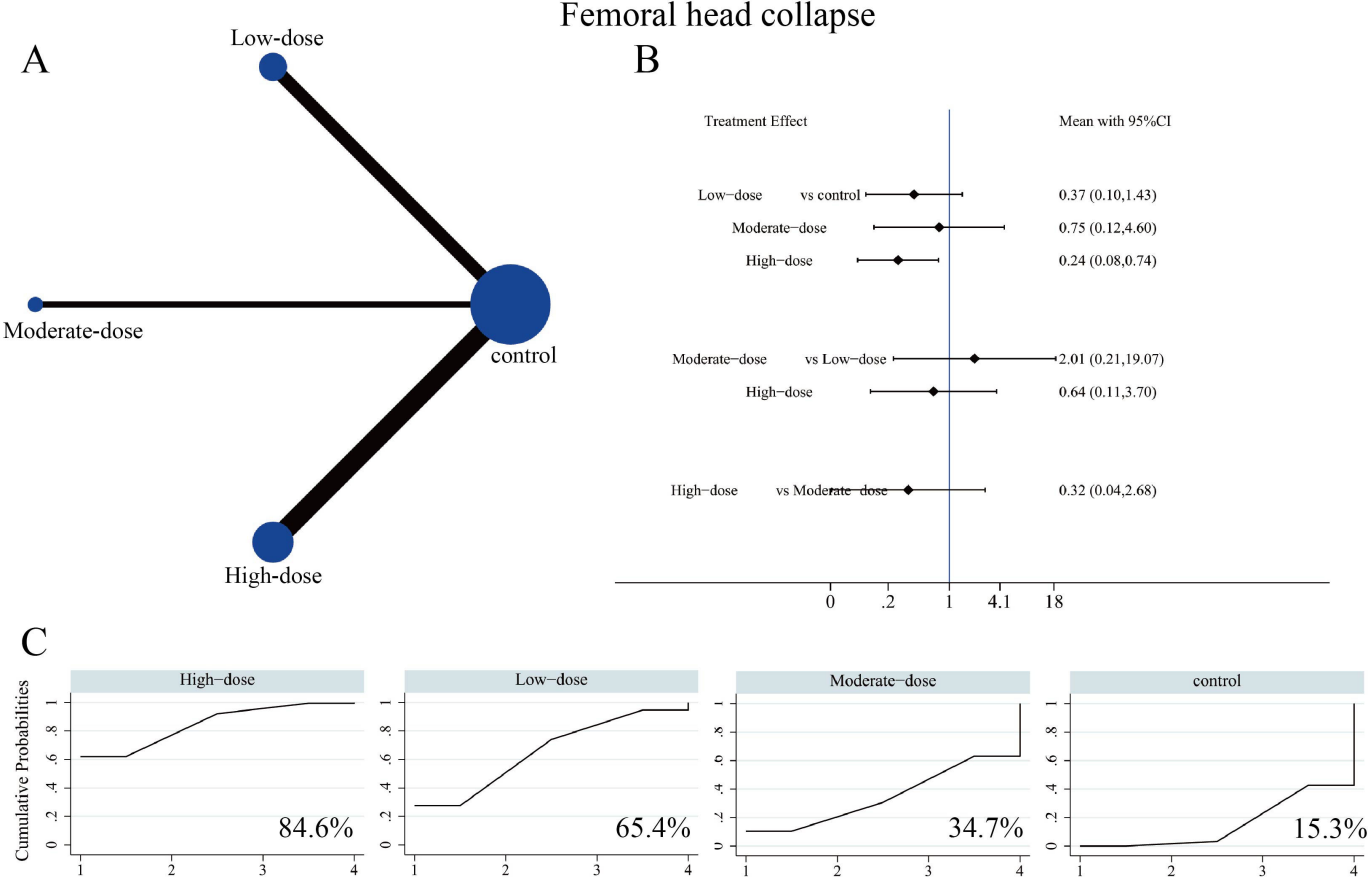
Network meta-analysis of the effects of different SC dosages on incidence of femoral head collapse. **(A)** Evidence network diagram for incidence of femoral head collapse. **(B)** Forest plot presenting comparative evidence of different SC dosages on incidence of femoral head. **(C)** SUCRA curves and surface under the cumulative ranking curve (%) for incidence of femoral head collapse after SC intervention.

Regarding THA conversion, 16 studies involving 1,125 patients reported clinical outcomes across the three SC dosage groups. The corresponding network evidence map is presented in **Figure 6A**. The network forest plot demonstrated that the high-dose SC group significantly reduced the rate of hip failure (OR = 0.28, 95% CI: 0.11-0.69) (**Figure 6B**, **Supplementary Table 2**). SUCRA values suggested that high-dose SC had the highest probability of being among the best-ranked strategies for reducing THA conversion (SUCRA 81%), followed by moderate-dose (63.4%) and low-dose (50.5%) (**Figure 6C**). For the incidence of femoral head collapse, 12 studies involving 857 patients were included. The network evidence map for the three SC dosage groups is presented in **Figure 7A**. The forest plot demonstrated that the high-dose SC group significantly reduced the incidence of femoral head collapse (OR = 0.24, 95% CI: 0.08-0.74) (**Figure 7B**, **Supplementary Table 3**), although no significant differences were detected among the three dosage groups. SUCRA suggested that high-dose SC had the highest probability of ranking best for reducing collapse (SUCRA 84.6%), followed by low-dose (65.4%), moderate-dose (34.7%), and control (15.3%) (**Figure 7C**). However, these rankings should be interpreted as probabilistic summaries rather than definitive evidence of superiority, given the overlapping CIs and limited precision for head-to-head dose comparisons.

#### VAS and WOMAC scores

**Figure 8:**
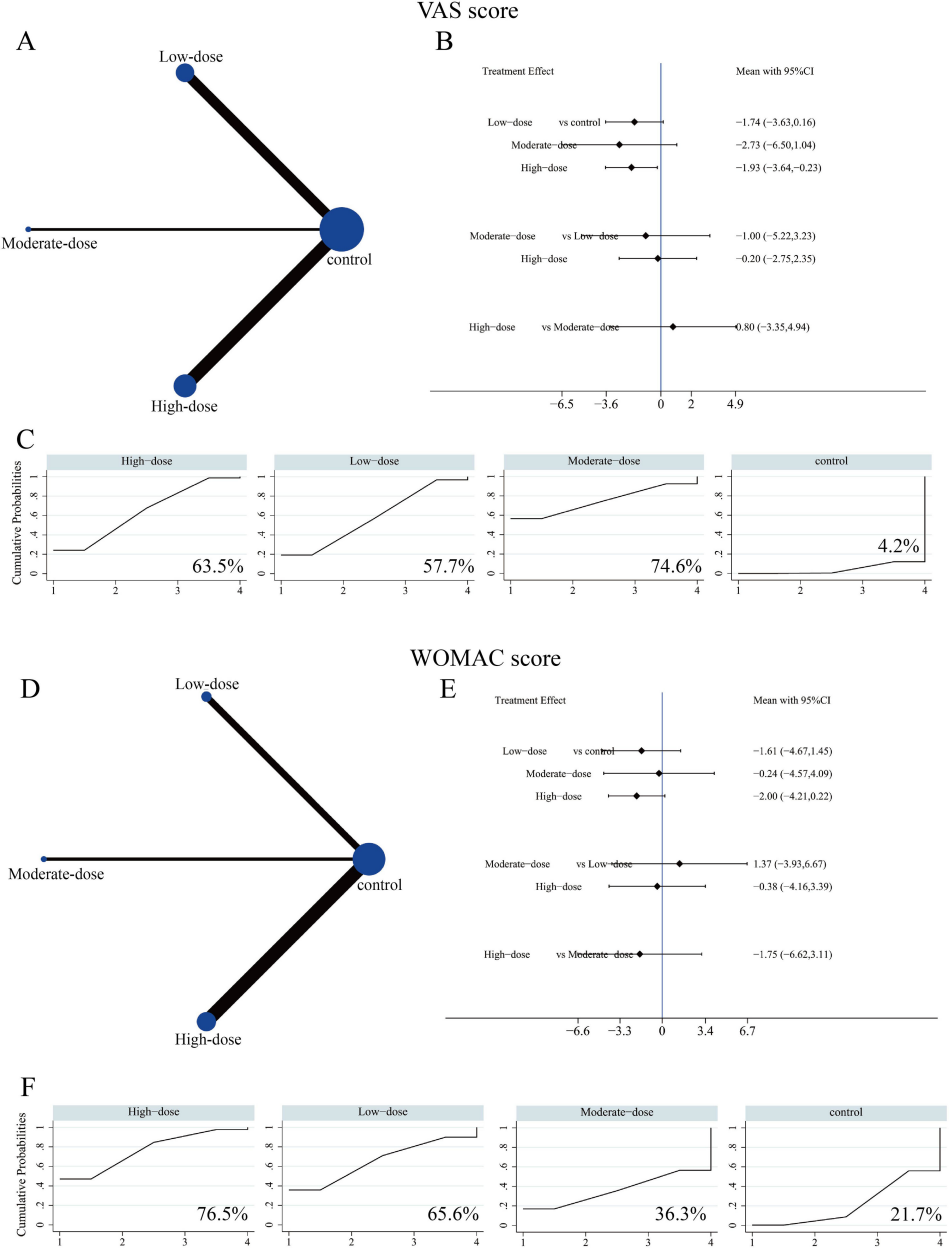
Network meta-analysis of the effects of different SC dosages on VAS and WOMAC scores. **(A)** Evidence network diagram of VAS score. **(B)** Forest plot presenting comparative evidence of different SC dosages on VAS score. **(C)** SUCRA curves and surface under the cumulative ranking curve (%) for VAS score after SC intervention. **(D)** Evidence network diagram of WOMAC score. **(E)** Forest plot presenting comparative evidence of different SC dosages on WOMAC score. **(F)** SUCRA curves and surface under the cumulative ranking curve (%) for WOMAC score after SC intervention.

In the NMA, VAS and WOMAC scores were used to assess pain relief and functional outcomes following SC treatment at different dosages. Ten studies involving 583 patients reported VAS scores. The network plot depicting high-, medium-, and low-dose groups is shown in **Figure 8A**. The forest plot demonstrated that the high-dose SC group significantly improved VAS scores compared with the control group (SMD = –1.93, 95% CI: –3.64 to –0.23), while no significant advantages were observed in the other dosage groups (**Figure 8B**, **Supplementary Table 4**). Based on SUCRA ranking, the medium-dose group showed the highest probability of being the most effective (74.6%), followed by the high-dose group (63.5%) and the low-dose group (57.7%) (**Figure 8C**).

For WOMAC scores, a total of seven studies involving 526 patients were included in the NMA. **Figure 8D** presents the network evidence map for the three different SC dosage groups. The analysis showed that SC treatment did not result in significant improvement in WOMAC scores across low-, medium-, or high-dose groups (**Figure 8E**, **Supplementary Table 5**), nor were there significant differences observed between dosage groups. SUCRA suggested that the high-dose group had the highest-ranking probability (76.5%), followed by the low-dose group (65.6%) (**Figure 8F**).

However, dose-to-dose comparisons were imprecise, and confidence intervals overlapped, SUCRA rankings should be interpreted as probabilistic summaries rather than definitive evidence of superiority.

#### Incidence of AEs

Additionally, we further compared the incidence of AEs following SC treatment at different dosages. Ten studies involving 519 patients reported AE incidence across the three SC dosage groups (**Supplementary Figure 8**). The network forest plot indicated no statistically significant differences among the dosage groups in reducing AE incidence (**Supplementary Table 6**). SUCRA values suggested that the high-dose group had the highest probability of ranking best (74.9%).

Based on the NMA and SUCRA findings, although the high-dose SC group did not exhibit a markedly superior effect compared with the low- and medium-dose groups, preliminary evidence suggests that high-dose SC may be beneficial in reducing rate of hip failure, incidence of hip collapse, and VAS score. However, given the limited number and sample sizes of pairwise comparisons, the interpretation of these results remains constrained.

Only one trial reported responder counts using a clinically important response, including radiographic non-progression and clinically relevant pain improvement. In Jayankura et al. (37), the 24-month composite treatment response (WOMAC pain improvement ≥10 mm plus no progression to fracture stage (ARCO ≥III)) was similar between SC plus CD and placebo plus CD (61% (14/23) vs 69% (18/26); *P* = 0.54). THA at 24 months was also similar (24% (6/25) vs 14% (4/29)). Given the small number of studies and inconsistent responder definitions, no meta-analysis was performed. Future trials should prespecify standardized MCID thresholds and report responder counts to enable quantitative synthesis.

#### Sensitivity analysis

Sensitivity analyses were conducted to assess the robustness of the conventional meta-analysis results (**Supplementary Figure 9**). For two key outcomes, including the rate of conversion to THA and femoral head collapse, the analyses showed that sequential exclusion of any single study did not materially alter the pooled results of the remaining studies, indicating that the summary findings for these outcomes were relatively stable. Additionally, given the heterogeneity in dose reporting and the concerns regarding dose ascertainment, we conducted an additional sensitivity analysis restricted to studies that explicitly reported quantitative total cell counts (**Supplementary Figure 10**). The pooled results for both conversion to THA and femoral head collapse were consistent with those of the primary analysis, with no material change in effect direction or statistical significance.

#### Publication bias

Additionally, potential publication bias was preliminarily assessed using funnel plots. For the conventional meta-analysis, no evident asymmetry was observed in the funnel plots, and this finding was further confirmed by Egger’s test (*P* > 0.05) (**Supplementary Figure 11**, **Table 3**). For the NMA, funnel plots related to key outcomes also showed no significant asymmetry (**Supplementary Figure 12**).

**Table 3:** Publication bias analysis for conversion to THA and femoral head collapse.

Outcome	Egger's test (p value)	t value	Pooling model
Rate of conversion to THA	0.696	-0.4	Random
Incidence of femoral head collapse	0.626	-0.5	Random

#### Certainty of evidence

According to the GRADE evaluation, the certainty of evidence for DFU healing rate was rated from moderate to very low, with very low certainty for the comparisons of emerging gel vs control, APG vs emerging gel, PDGF gel vs mature gel, PDGF gel vs emerging gel, and mature gel vs emerging gel. The certainty of evidence for complete healing time was rated as low to very low (**Supplementary Table 7**). For the incidence of AEs, the certainty ranged from moderate to very low, with very low certainty for APG vs mature gel. The certainty of evidence for the incidence of tissue infection and SAEs was rated as moderate to low (**Supplementary Table 4**). The judgments of lower certainty were mainly attributed to concerns about high risk of bias, substantial heterogeneity, or imprecision in several comparisons. Therefore, although the outcomes of healing rate and AE incidence are clinically important, the current evidence does not allow definitive conclusions regarding the true effectiveness of gel-assisted therapy for DFU.

The results based on the GRADE assessment indicate that the certainty of evidence for conversion to THA ranges from moderate to very low. Except for the comparison between the high-dose group and the control group, which was assessed as having moderate certainty, all other five comparisons were considered to have very low certainty. For femoral head collapse, the certainty of evidence for all comparison groups was very low, mainly due to high risk of bias, significant heterogeneity, and concerns about the imprecision in multiple comparisons (**Supplementary Figure 7**). Therefore, a clear conclusion regarding the effect of high-dose SC combined with CD in reducing hip failure rates in ONFH patients cannot be drawn at present and requires further validation.

## Discussion

The main finding of this systematic review and NMA is that high-dose autologous SC (>1×10^8) combined with CD for the treatment of ONFH demonstrated promising therapeutic potential across several key outcomes, including significantly decreasing hip failure rates, lowering the risk of hip collapse, and reducing pain as measured by VAS scores. Although direct comparisons did not consistently reveal significant statistical superiority over low- and medium-dose groups, the SUCRA ranking results consistently suggested that the high-dose group may perform best in several outcomes, such as lowering hip failure and femoral head collapse. However, given the presence of a certain degree of heterogeneity among the included studies and the limited number of subgroup analyses, the current evidence is insufficient to definitively support high-dose SC as the absolute optimal strategy. Further high-quality studies are warranted for validation.

CD is a well-established surgical approach for the treatment of osteonecrosis of the femoral head, promoting bone regeneration by reducing intraosseous pressure and increasing blood flow to the necrotic area (52). However, the clinical benefits of CD at present remain limited and often unsatisfactory, particularly in patients with ARCO stage III and IV disease (53). Owing to the pathological characteristics of ONFH and the secretory and trophic functions of stem cells, stem cell therapy combined with CD has been increasingly applied in clinical practice, showing positive effects in reducing the risk of femoral head collapse and accelerating bone reconstruction within necrotic regions (54, 55). In a previous meta-analysis, Zhang et al. (19) found that BMSC intervention resulted in better clinical outcomes and a lower disease progression rate compared to CD alone. Our study further refined the evaluation of combined therapy by examining the impact of stem cell dosage and found that high-dose SC (>1×10^8) may achieve superior outcomes across several key indicators, including rate of conversion to THA, incidence of femoral head collapse, and VAS score. This finding supplements the overall effectiveness conclusions of previous meta-analyses by providing evidence at the dose–response level. Four prior meta-analyses consistently demonstrated that SC/BMSC combined with CD significantly delayed disease progression, reduced arthroplasty rates, and improved pain and functional scores compared with CD alone (17–20). Our study further suggests that these advantages may, to some extent, be associated with stem cell dosage, as the high-dose group showed more pronounced clinical benefits across multiple outcomes. However, due to the limited number of direct comparisons among different dosage groups in existing studies, along with heterogeneity and variations in follow-up duration, it remains difficult to establish a definitive dose–response relationship. In addition, although the combined therapy group overall demonstrated good safety, our study found no significant differences in adverse event rates across different SC dosage groups, which may suggest that treatment within an effective dosage range remains safe. In summary, current evidence supports the overall value of SC combined with CD in the management of early-stage ONFH. The dose-based subgroup analyses and NMA in this study provide preliminary guidance for optimizing this combined strategy, though further high-quality research is needed to determine the optimal cell dosage and long-term efficacy.

### Optimal cell counts for bone tissue repair

Although previous studies have confirmed the effectiveness of cell therapy combined with CD, the optimal stem cell dose remains a critical issue to be addressed in both clinical practice and research. Based on the b0069xological characteristics of a normal femoral head, it contains approximately 35,000 MSCs, a value commonly regarded as a reference quantity required for bone repair (56). In patients with osteonecrosis of the femoral head, the number of MSCs within the necrotic region is markedly reduced or even absent, while the surrounding areas are also affected, resulting in an overall depletion of the cellular reservoir (16, 57). Theoretical estimations suggest that the number of implanted cells should be adjusted according to the necrotic lesion volume; for instance, in a lesion involving roughly one-third of the femoral head (approximately 17 cm³), about 12,000 MSCs would theoretically be needed (56). However, since MSC levels in the peri-necrotic area are usually diminished, the actual number of required cells is expected to exceed this theoretical value.

However, clinical evidence indicates that the relationship between MSC counts and repair efficacy is not strictly linear (58, 59). When the number of transplanted cells increases to the million level, repair volume shows a certain correlation with cell counts, but beyond a threshold the therapeutic improvement tends to plateau, or may even slightly decline. This suggests that excessively high doses may not provide additional benefits and could, in fact, reduce efficiency due to cell loss or limitations within the local microenvironment. Our network meta-analysis results support this perspective: although the high-dose group demonstrated relative advantages in reducing femoral head collapse and lowering conversion rates to THA, direct comparisons between different dosage groups did not always yield consistent results, highlighting the uncertainty surrounding the dose–response relationship.

Furthermore, the actual retention rate of implanted cells significantly influences efficacy. Studies have shown that only 30%–50% of labeled cells remain in the femoral head within 24 hours after injection (60). Cell loss is primarily related to venous drainage, and there is a close relationship between injection volume and retention rate: an excessively large injection volume may increase cell loss, while an overly small volume may fail to adequately fill the necrotic region, thereby compromising repair outcomes (31). Thus, determining the optimal cell dose requires integrated consideration of lesion volume, cell retention efficiency, and microenvironmental factors. Our findings suggest that high-dose transplantation (>1×10^8) tends to achieve superior outcomes in terms of pain relief, reduced incidence of femoral head collapse, and lower THA rates. Although the absolute values differ from the theoretically derived requirement of 24,000–35,000 cells, the results underscore the critical role of ensuring sufficient cell numbers to achieve biological repair. Future research should incorporate imaging-guided techniques, monitoring of cell retention, and patient-specific factors to more precisely define the optimal cell dosage for different clinical scenarios.

### AEs and cell counts

In addition to investigating the beneficial effects of high-dose SC on symptom relief and disease progression in patients with osteonecrosis of the femoral head, it is equally important to evaluate the potential AEs associated with high-dose SC, as this is critical for informing and guiding clinical decision-making. Overall, previous studies have reported good safety profiles for stem cell therapy, with a relatively low incidence of AEs, most of which were mild and self-limiting, including donor site pain, hematoma, infection, and deep vein thrombosis (61–63). For example, several meta-analyses have shown no significant differences in complication rates between SC combined with CD and CD alone (17, 59). However, these studies did not directly analyze the association between AEs and stem cell dosage, and the small number of events limited their statistical power. In our study, the network meta-analysis further indicated that no significant differences in AE incidence were observed among the low-, medium-, and high-dose SC groups. SUCRA ranking suggested that the high-dose group might have the best safety profile (74.9%), but this conclusion was derived from limited data (only five studies included in the pooled analysis) with considerable heterogeneity, and thus should be interpreted cautiously. These findings suggest that, based on current evidence, increasing stem cell dosage may not markedly elevate the risk of AEs. Nevertheless, due to restrictions in sample size and follow-up duration, the long-term safety of high-dose SC requires further validation through high-quality studies. Future research should emphasize standardized reporting of AEs and stratified analyses by dosage to more precisely assess the impact of cell counts on safety.

## Limitations

To the best of our knowledge, this meta-analysis aims to evaluate the clinical efficacy of SC combined with CD for osteonecrosis of the femoral head from the perspective of different cell dosages. Despite a comprehensive synthesis of the available evidence, several limitations should be acknowledged when interpreting the findings. Firstly, significant heterogeneity was observed among the included studies in terms of patient characteristics (e.g., etiology and stage of osteonecrosis), cell processing protocols, and the dosage stratification standards applied in subgroup analyses. Although random-effects models and subgroup analyses were employed, inherent clinical and methodological differences may still have influenced the pooled effect estimates. Secondly, the classification of low-, medium-, and high-dose stem cell groups was based on a limited number of studies, with some subgroups including only a single trial. This may reduce the reliability of dose-dependent comparisons. Furthermore, the network meta-analysis relied in part on indirect comparisons, although statistical approaches were used to ensure consistency, indirect evidence may introduce additional uncertainty compared with direct comparisons. Given the heterogeneity of cell preparations and counting methods, our results may not be directly generalizable across different product types at the same dose.

## Conclusion

This systematic review and NMA indicated that high-dose autologous stem cell therapy (>1×10^8 cells) combined with CD is associated with a lower risk of hip failure and a lower femoral head collapse rate compared with CD alone. Improvements in pain and function outcomes were also observed in some comparisons, whereas no clear differences in AE rates were detected across dose categories. However, due to the substantial heterogeneity of included studies and limited sample sizes, these conclusions should be interpreted with caution. Further high-quality research is needed to validate the optimal dosage threshold and long-term efficacy.

## Data Availability

The original contributions presented in the study are included in the article/**Supplementary Material**. Further inquiries can be directed to the corresponding author.
