# Supplementary material for: Efficacy of autologous stem cell at different doses combined with core decompression in the treatment of osteonecrosis of the femoral head: a systematic review and network meta-analysis
Source: Front Endocrinol (Lausanne). 2026 Apr 22;17:1720437. doi: 10.3389/fendo.2026.1720437 (PMC13143691; doi:10.3389/fendo.2026.1720437)
Supplement: Supplementary file 1 [file DataSheet1.pdf]

Supplementary Materials

Search strategies in different databases:

Pubmed (Search Cut-off Date: July 1, 2025)

#1 “Mesenchymal Stem Cells” OR “Mesenchymal Stem Cell” OR “Bone Marrow Mesenchymal Stem Cells” OR “Bone Marrow Mesenchymal Stem Cell” OR “Bone Marrow Stromal Cells” OR “Bone Marrow Stromal Cell” OR “Multipotent Bone Marrow Stromal Cell” OR “Multipotent Bone Marrow Stromal Cells” OR “Adipose-Derived Mesenchymal Stem Cells” OR “Adipose Derived Mesenchymal Stem Cells” OR “Adipose-Derived Mesenchymal Stromal Cells” OR “Adipose Derived Mesenchymal Stromal Cells” OR “Adipose-Derived Mesenchymal Stem Cell” OR “Adipose Derived Mesenchymal Stem Cell” OR “Adipose Tissue-Derived Mesenchymal Stem Cell” OR “Adipose Tissue Derived Mesenchymal Stem Cell” OR “Adipose Tissue-Derived Mesenchymal Stem Cells” OR “Adipose Tissue Derived Mesenchymal Stem Cells” OR “Adipose Tissue-Derived Mesenchymal Stromal Cells” OR “Adipose Tissue Derived Mesenchymal Stromal Cells” OR “Adipose Tissue-Derived Mesenchymal Stromal Cell” OR “Adipose Tissue Derived Mesenchymal Stromal Cell” OR “Mesenchymal Stromal Cells” OR “Mesenchymal Stromal Cell” OR “Stromal Cells, Mesenchymal” OR “Multipotent Mesenchymal Stromal Cells” OR “Multipotent Mesenchymal Stromal Cell” OR “Mesenchymal Progenitor Cell” OR “Mesenchymal Progenitor Cells” OR “Wharton Jelly Cells” OR “Wharton's Jelly Cells” OR “Wharton's Jelly Cell” OR “Whartons Jelly Cells” OR “Bone Marrow Stromal Stem Cells” OR “Stem Cells” OR “Stem Cell” OR “Progenitor Cells” OR “Progenitor Cell” OR “Mother Cells” OR “Mother Cell”

Results: 158,027

#2 “Osteonecrosis” OR “Osteonecroses” OR “Bone Necrosis” OR “Bone Necroses” OR “Avascular Necrosis of Bone” OR “Bone Avascular Necrosis” OR “Kienbock Disease” OR “Kienbock's Disease” OR “Kienboeck Disease” OR “Kienboeck's Disease” OR “Kienboecks Disease” OR “Aseptic Necrosis of Bone” OR “Bone Aseptic Necrosis” AND “Femur Head” OR “Femur Heads” OR “Femoral Head” OR “Femoral Heads”

Results: 22,097

#3 “Core decompression” OR “CD”

Results: 1328

#4 “Controlled clinical trial” OR “Randomized controlled trial” OR “RCT”

Results: 825,423

#5 #1 AND #2 AND #3 AND #4

Results: 14

Cochrane Library (Search Cut-off Date: July 1, 2025)

#6 (Mesenchymal Stem Cells):ab,ti,kw OR (Mesenchymal Stem Cell):ab,ti,kw OR (Bone Marrow Mesenchymal Stem Cells):ab,ti,kw OR (Bone Marrow Mesenchymal Stem Cell):ab,ti,kw OR (Bone Marrow Stromal Cells):ab,ti,kw OR (Bone Marrow Stromal Cell):ab,ti,kw OR (Multipotent Bone Marrow Stromal Cell):ab,ti,kw OR (Multipotent Bone Marrow Stromal Cells):ab,ti,kw OR (Adipose-Derived Mesenchymal Stem Cells):ab,ti,kw OR (Adipose Derived Mesenchymal Stem Cells):ab,ti,kw OR (Adipose-Derived Mesenchymal Stromal Cells):ab,ti,kw OR (Adipose Derived Mesenchymal Stromal Cells):ab,ti,kw OR (Adipose-Derived Mesenchymal Stem Cell):ab,ti,kw OR (Adipose Derived Mesenchymal Stem Cell):ab,ti,kw OR (Adipose Tissue-Derived Mesenchymal Stem Cell):ab,ti,kw OR (Adipose Tissue Derived Mesenchymal Stem Cell):ab,ti,kw OR (Adipose

Tissue-Derived Mesenchymal Stem Cells):ab,ti,kw OR (Adipose Tissue Derived Mesenchymal Stem Cells):ab,ti,kw OR (Adipose Tissue-Derived Mesenchymal Stromal Cells):ab,ti,kw OR (Adipose Tissue Derived Mesenchymal Stromal Cells):ab,ti,kw OR (Adipose Tissue-Derived Mesenchymal Stromal Cell):ab,ti,kw OR (Adipose Tissue Derived Mesenchymal Stromal Cell):ab,ti,kw OR (Mesenchymal Stromal Cells):ab,ti,kw OR (Mesenchymal Stromal Cell):ab,ti,kw OR (Stromal Cells, Mesenchymal):ab,ti,kw OR (Multipotent Mesenchymal Stromal Cells):ab,ti,kw OR (Multipotent Mesenchymal Stromal Cell):ab,ti,kw OR (Mesenchymal Progenitor Cell):ab,ti,kw OR (Mesenchymal Progenitor Cells):ab,ti,kw OR (Wharton Jelly Cells):ab,ti,kw OR (Wharton's Jelly Cells):ab,ti,kw OR (Wharton's Jelly Cell):ab,ti,kw OR (Whartons Jelly Cells):ab,ti,kw OR (Bone Marrow Stromal Stem Cells):ab,ti,kw OR (Stem Cells):ab,ti,kw OR (Stem Cell):ab,ti,kw OR (Progenitor Cells):ab,ti,kw OR (Progenitor Cell):ab,ti,kw OR (Mother Cells):ab,ti,kw OR (Mother Cell):ab,ti,kw

Results: 19,480

#7 (Osteonecrosis):ab,ti,kw OR (Osteonecroses):ab,ti,kw OR (Bone Necrosis):ab,ti,kw OR (Bone Necroses):ab,ti,kw OR (Avascular Necrosis of Bone):ab,ti,kw OR (Bone Avascular Necrosis):ab,ti,kw OR (Kienbock Disease):ab,ti,kw OR (Kienbock's Disease):ab,ti,kw OR (Kienboeck Disease):ab,ti,kw OR (Kienboeck's Disease):ab,ti,kw OR (Kienboecks Disease):ab,ti,kw OR (Aseptic Necrosis of Bone):ab,ti,kw OR (Bone Aseptic Necrosis):ab,ti,kw AND (Femur Head):ab,ti,kw OR (Femur Heads):ab,ti,kw OR (Femoral Head):ab,ti,kw OR (Femoral Heads):ab,ti,kw

Results: 3,502

#8 (Core decompression):ab,ti,kw OR (CD):ab,ti,kw

Results: 3

#9 (Controlled clinical trial):ab,ti,kw OR (randomized controlled trial):ab,ti,kw OR (RCT):ab,ti,kw

Results: 875,347

#10 #6 AND #7 AND #8 AND #9

Results: 75

Web of Science (Search Cut-off Date: July 1, 2025)

#11 TS=(Mesenchymal Stem Cells OR Mesenchymal Stem Cell OR Bone Marrow Mesenchymal Stem Cells OR Bone Marrow Mesenchymal Stem Cell OR Bone Marrow Stromal Cells OR Bone Marrow Stromal Cell OR Multipotent Bone Marrow Stromal Cell OR Multipotent Bone Marrow Stromal Cells OR Adipose-Derived Mesenchymal Stem Cells OR Adipose Derived Mesenchymal Stem Cells OR Adipose-Derived Mesenchymal Stromal Cells OR Adipose Derived Mesenchymal Stromal Cells OR Adipose-Derived Mesenchymal Stem Cell OR Adipose Derived Mesenchymal Stem Cell OR Adipose Tissue-Derived Mesenchymal Stem Cell OR Adipose Tissue Derived Mesenchymal Stem Cell OR Adipose Tissue-Derived Mesenchymal Stem Cells OR Adipose Tissue Derived Mesenchymal Stem Cells OR Adipose Tissue-Derived Mesenchymal Stromal Cells OR Adipose Tissue Derived Mesenchymal Stromal Cells OR Adipose Tissue-Derived Mesenchymal Stromal Cell OR Adipose Tissue Derived Mesenchymal Stromal Cell OR Mesenchymal Stromal Cells OR Mesenchymal Stromal Cell OR Stromal Cells, Mesenchymal OR Multipotent Mesenchymal Stromal Cells OR Multipotent Mesenchymal Stromal Cell OR Mesenchymal Progenitor Cell OR Mesenchymal Progenitor Cells OR Wharton Jelly Cells OR Wharton's Jelly Cells OR Wharton's Jelly Cell OR Whartons Jelly Cells OR Bone Marrow Stromal Stem Cells OR

Stem Cells OR Stem Cell OR Progenitor Cells OR Progenitor Cell OR Mother Cells OR Mother Cell)
Results: 298,202
#12 TS=(Osteonecrosis OR Osteonecroses OR Bone Necrosis OR Bone Necroses OR Avascular Necrosis of Bone OR Bone Avascular Necrosis OR Kienbock Disease OR Kienbock's Disease OR Kienboeck Disease OR Kienboeck's Disease OR Kienboecks Disease OR Aseptic Necrosis of Bone OR Bone Aseptic Necrosis AND Femur Head OR Femur Heads OR Femoral Head OR Femoral Heads)
Results: 19,637
#13 TS=(Core decompression OR CD)
Results: 2367
#14 TS=(Controlled clinical trial OR randomized controlled trial OR RCT)
Results: 304,803
#15 #11 AND #12 AND #13 AND #14
Results: 68

Embase (Search Cut-off Date: July 1, 2025)
#16 (‘Mesenchymal Stem Cells’:ab,ti,kw OR ‘Mesenchymal Stem Cell’:ab,ti,kw OR ‘Bone Marrow Mesenchymal Stem Cells’:ab,ti,kw OR ‘Bone Marrow Mesenchymal Stem Cell’:ab,ti,kw OR ‘Bone Marrow Stromal Cells’:ab,ti,kw OR ‘Bone Marrow Stromal Cell’:ab,ti,kw OR ‘Multipotent Bone Marrow Stromal Cell’:ab,ti,kw OR ‘Multipotent Bone Marrow Stromal Cells’:ab,ti,kw OR ‘Adipose-Derived Mesenchymal Stem Cells’:ab,ti,kw OR ‘Adipose Derived Mesenchymal Stem Cells’:ab,ti,kw OR ‘Adipose-Derived Mesenchymal Stromal Cells’:ab,ti,kw OR ‘Adipose Derived Mesenchymal Stromal Cells’:ab,ti,kw OR ‘Adipose-Derived Mesenchymal Stem Cell’:ab,ti,kw OR ‘Adipose Derived Mesenchymal Stem Cell’:ab,ti,kw OR ‘Adipose Tissue-Derived Mesenchymal Stem Cell’:ab,ti,kw OR ‘Adipose Tissue Derived Mesenchymal Stem Cell’:ab,ti,kw OR ‘Adipose Tissue-Derived Mesenchymal Stem Cells’:ab,ti,kw OR ‘Adipose Tissue Derived Mesenchymal Stem Cells’:ab,ti,kw OR ‘Adipose Tissue-Derived Mesenchymal Stromal Cells’:ab,ti,kw OR ‘Adipose Tissue Derived Mesenchymal Stromal Cells’:ab,ti,kw OR ‘Adipose Tissue-Derived Mesenchymal Stromal Cell’:ab,ti,kw OR ‘Adipose Tissue Derived Mesenchymal Stromal Cell’:ab,ti,kw OR ‘Mesenchymal Stromal Cells’:ab,ti,kw OR ‘Mesenchymal Stromal Cell’:ab,ti,kw OR ‘Stromal Cells, Mesenchymal’:ab,ti,kw OR ‘Multipotent Mesenchymal Stromal Cells’:ab,ti,kw OR ‘Multipotent Mesenchymal Stromal Cell’:ab,ti,kw OR ‘Mesenchymal Progenitor Cell’:ab,ti,kw OR ‘Mesenchymal Progenitor Cells’:ab,ti,kw OR ‘Wharton Jelly Cells’:ab,ti,kw OR ‘Wharton Jelly Cells’:ab,ti,kw OR ‘Wharton Jelly Cell’:ab,ti,kw OR ‘Whartons Jelly Cells’:ab,ti,kw OR ‘Bone Marrow Stromal Stem Cells’:ab,ti,kw OR ‘Stem Cells’:ab,ti,kw OR ‘Stem Cell’:ab,ti,kw OR ‘Progenitor Cells’:ab,ti,kw OR ‘Progenitor Cell’:ab,ti,kw OR ‘Mother Cells’:ab,ti,kw OR ‘Mother Cell’:ab,ti,kw)
Results: 676,832
#17 (‘Osteonecrosis’:ab,ti,kw OR ‘Osteonecroses’:ab,ti,kw OR ‘Bone Necrosis’:ab,ti,kw OR ‘Bone Necroses’:ab,ti,kw OR ‘Avascular Necrosis of Bone’:ab,ti,kw OR ‘Bone Avascular Necrosis’:ab,ti,kw OR ‘Kienbock Disease’:ab,ti,kw OR ‘Kienboeck Disease’:ab,ti,kw OR ‘Kienboecks Disease’:ab,ti,kw OR ‘Aseptic Necrosis of Bone’:ab,ti,kw OR ‘Bone Aseptic Necrosis’:ab,ti,kw AND ‘Femur Head’:ab,ti,kw OR ‘Femur Heads’:ab,ti,kw OR ‘Femoral

Head’:ab,ti,kw OR ‘Femoral Heads’:ab,ti,kw)
Results: 27,950
#18 ('Core decompression':ab,ti,kw OR 'CD':ab,ti,kw)
Results: 295,43
#19 ('Controlled clinical trial':ab,ti,kw OR 'randomized controlled trial':ab,ti,kw OR 'RCT':ab,ti,kw)
Results: 305,75
#20 #16 AND #17 AND #18 AND #19
Results: 9

CNKI (Search Cut-off Date: July 1, 2025)
#21 (SU='股骨头坏死' OR SU='股骨头缺血性坏死' OR SU='ONFH' OR SU='AVNFH')
Results: 16,693
#22 (SU='干细胞' OR SU='间充质干细胞' OR SU='MSC' OR SU='BMSC' OR SU='骨髓基质细胞' OR SU='祖细胞' OR SU='基质血管成分' OR SU='SVF' OR SU='细胞移植' OR SU='细胞治疗')
Results: 187,134
#23 (SU='钻孔减压术' OR SU='CD')
Results: 210
#24 (SU='随机对照试验' OR SU='RCT')
Results: 23,794
#25 #21 AND #22 AND #23 AND #24
Results: 2
#26 #5 AND #10 AND #15 AND #20 AND #25
Results: 168

Supplementary Table S1. Newcastle-Ottawa Scale for risk of bias assessment of retrospective studies included in the meta-analysis.

Study	Selection				Comparability	Exposure			Scores
	Representativeness of Exposed Cohort	Selection of Nonexposed	Ascertainment of Exposure	Outcome Not Present at Start	Comparability between groups	Assessment of Outcome	Adequate Follow-Up Length	Adequacy of Follow-Up	
Gangji et al 2011	0.5	1	1	1	1.5	1	1	0	7
Hernigou et al 2018	0.5	1	1	1	2	1	1	1	8.5
Hoogervorst et al 2022	0.5	0.5	1	0	1	1	1	1	6
Kang et al 2018	0.5	0.5	0.5	0	2	0.5	1	0	5

Supplementary Table S2. League table demonstrating the results of the network meta-analysis comparing the rate of conversion to THA (OR, 95% CI).

Treatment	High-dose	Moderate-dose	Low-dose	control
High-dose	1	1.36 (0.31,5.92)	1.72 (0.40,7.49)	**3.60 (1.44,8.97)**
Moderate-dose	0.73 (0.17,3.19)	1	1.27 (0.25,6.47)	2.64 (0.84,8.35)
Low-dose	0.58 (0.13,2.52)	0.79 (0.15,4.04)	1	2.09 (0.66,6.57)
control	**0.28 (0.11,0.69)**	0.38 (0.12,1.20)	0.48 (0.15,1.51)	1

OR: odds ratio; THA: total hip arthroplasty.

Supplementary Table S3. League table demonstrating the results of the network meta-analysis comparing the incidence of femoral head collapse (OR, 95% CI).

Treatment	High-dose	Low-dose	Moderate-dose	control
High-dose	1	1.56 (0.27,8.94)	3.13 (0.37,26.22)	**4.15 (1.35,12.77)**
Low-dose	0.64 (0.11,3.70)	1	2.01 (0.21,19.07)	2.67 (0.70,10.17)
Moderate-dose	0.32 (0.04,2.68)	0.50 (0.05,4.72)	1	1.33 (0.22,8.11)
control	**0.24 (0.08,0.74)**	0.37 (0.10,1.43)	0.75 (0.12,4.60)	1

OR: odds ratio.

Supplementary Table S4. League table demonstrating the results of the network meta-analysis comparing the VAS (SMD, 95% CI).

Treatment	Moderate-dose	High-dose	Low-dose	control
Moderate-dose	1	0.80 (-3.35,4.94)	1.00 (-3.23,5.22)	2.73 (-1.04,6.50)
High-dose	-0.80 (-4.94,3.35)	1	0.20 (-2.35,2.75)	**1.93 (0.23,3.64)**
Low-dose	-1.00 (-5.22,3.23)	-0.20 (-2.75,2.35)	1	1.74 (-0.16,3.63)
control	-2.73 (-6.50,1.04)	**-1.93 (-3.64,-0.23)**	-1.74 (-3.63,0.16)	1

SMD: standardized mean difference; VAS: visual analogue scale.

Supplementary Table S5. League table demonstrating the results of the network meta-analysis comparing the WOMAC score (SMD, 95% CI).

Treatment	High-dose	Low-dose	Moderate-dose	control
High-dose	1	1.47 (0.03,64.04)	5.76 (0.04,746.64)	7.36 (0.80,67.47)
Low-dose	0.68 (0.02,29.73)	1	3.93 (0.02,786.98)	5.01 (0.24,106.58)
Moderate-dose	0.17 (0.00,22.48)	0.25 (0.00,51.04)	1	1.28 (0.02,96.93)
control	0.14 (0.01,1.25)	0.20 (0.01,4.24)	0.78 (0.01,59.50)	1

SMD: standardized mean difference; WOMAC: Western Ontario and McMaster Universities Osteoarthritis Index.

Supplementary Table S6. League table demonstrating the results of the network meta-analysis comparing the incidence of AE (OR, 95% CI).

Treatment	High-dose	Low-dose	Moderate-dose	control

High-dose	1	1.70 (0.20,14.17)	2.02 (0.26,15.82)	2.05 (0.39,10.84)
Low-dose	0.59 (0.07,4.93)	1	1.19 (0.20,7.11	1.21 (0.32,4.51)
Moderate-dose	0.49 (0.06,3.86)	0.84 (0.14,4.99)	1	1.01 (0.30,3.38)
control	0.49 (0.09,2.58)	0.83 (0.22,3.09)	0.99 (0.30,3.30)	1

AE: adverse event; OR: odds ratio.

Supplementary Table S7. Direct and network meta-analysis evidence and GRADE ratings for comparison of different SC dosages on conversion to THA and femoral head collapse.

Outcome	Comparison	`	NMA estimate	Certainty in the Evidence
		OR (95% CI)	OR (95% CI)	
	Low-dose vs. control	0.49 (0.07 to 3.37)	0.48 (0.15 to 1.51)	⊕ very low[a,b,c,d]
	Moderate-dose vs. control	0.38 (0.11 to 1.23)	0.38 (0.12 to 1.20)	⊕ very low[a,b,c,d]
**Conversion to THA**	High-dose vs. control	0.28 (0.15 to 0.51)	0.28 (0.11 to 0.69)	⊕⊕⊕ Moderate[c]
	Low-dose vs. Moderate-dose	/	0.79 (0.15 to 4.04)	⊕ very low[a,c,d]
	Moderate-dose vs. High-dose	/	0.73 (0.17 to 3.19)	⊕ very low[a,c,d]

	Low-dose vs. High-dose	/	0.58 (0.13 to 2.52)	⊕ very low[a,c,d]
Femoral head collapse	Low-dose vs. control	0.37 (0.07 to 2.11)	0.37 (0.10 to 1.43)	⊕ very low[a,b,c,d]
	Moderate-dose vs. control	0.81 (0.32 to 2.10)	0.75 (0.12 to 4.60)	⊕ very low[a,c,d]
	High-dose vs. control	0.25 (0.09 to 0.68)	0.24 (0.08 to 0.74)	⊕ very low[a,b,c]
	Low-dose vs. Moderate-dose	/	2.01 (0.21 to 19.07)	⊕ very low[a,c,d]
	Moderate-dose vs. High-dose	/	0.32 (0.04 to 2.68)	⊕ very low[a,c,d]
	Low-dose vs. High-dose	/	0.64 (0.11 to 3.70)	⊕ very low[a,c,d]

CI: confidence interval; NMA: network meta-analysis; OR: odds ratio; SC: Stem cell; THA: total hip arthroplasty.⨁⨁⨁⨁: high certainty; ⨁⨁⨁: moderate certainty; ⨁⨁: low certainty; ⨁: very low certainty; a. Certainty in the evidence downgraded by one level due to significant risk of bias; b. Certainty in the evidence downgraded by one level due to inconsistency; c. Certainty in the evidence downgraded by one level due to significant publication bias; d. Certainty in the evidence downgraded by one level due to imprecision.

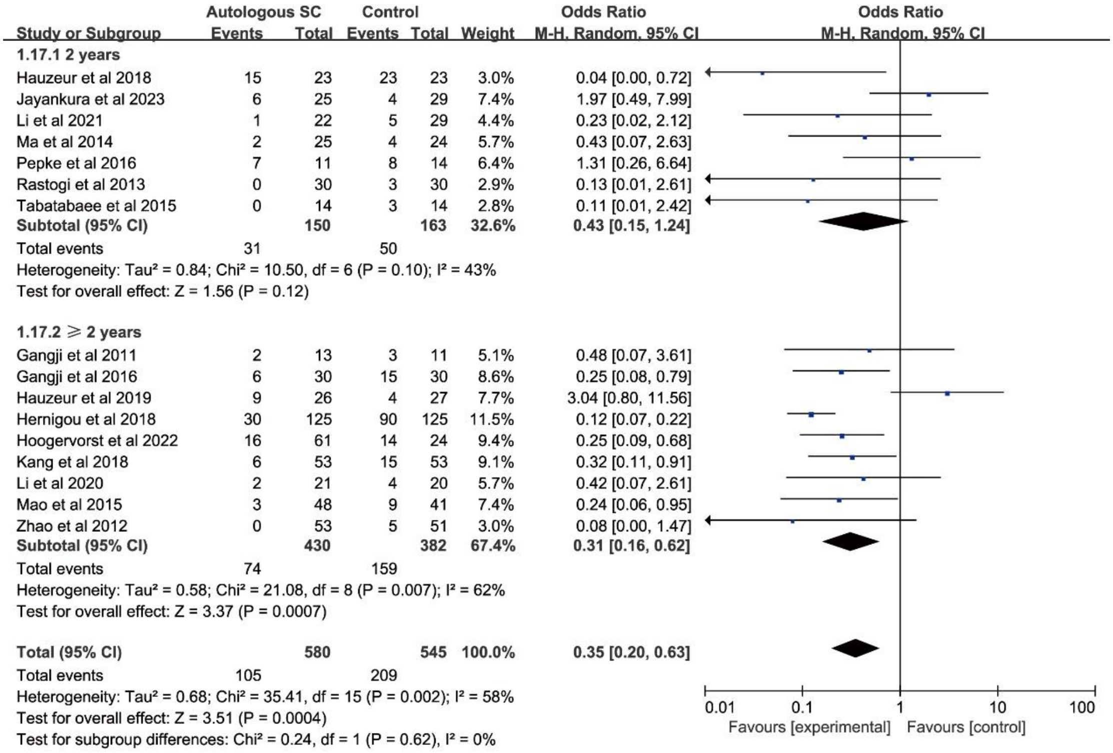

Supplementary Figure S1. Forest plot illustrating the effects of SC on conversion to THA based on different follow-up durations.

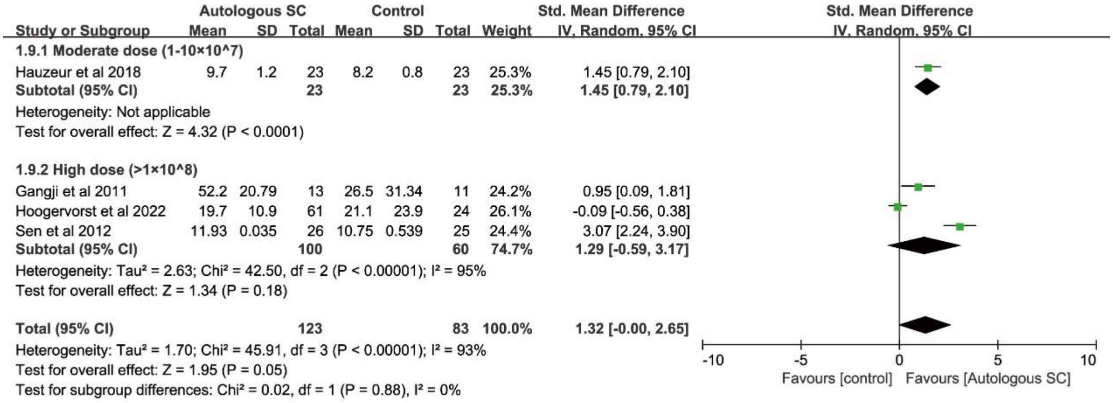

Supplementary Figure S2. Forest plot illustrating the effects of different SC dosages on average survival time of hip.

Study or Subgroup	Autologous SC Events	Autologous SC Total	Control Events	Control Total	Weight	Odds Ratio M-H, Random, 95% CI
**1.19.1 2 years**						
Chang et al 2010	0	8	1	8	3.5%	0.29 [0.01, 8.37]
Hauzeur et al 2018	10	23	9	23	9.8%	1.20 [0.37, 3.88]
Ma et al 2014	2	25	8	24	7.7%	0.17 [0.03, 0.93]
Rastogi et al 2013	12	30	12	30	10.4%	1.00 [0.36, 2.81]
Tabatabaee et al 2015	0	14	11	14	3.9%	0.01 [0.00, 0.22]
**Subtotal (95% CI)**		**100**		**99**	**35.2%**	**0.36 [0.10, 1.36]**
Total events	24		41			
Heterogeneity: Tau² = 1.35; Chi² = 11.85, df = 4 (P = 0.02); I² = 66%						
Test for overall effect: Z = 1.51 (P = 0.13)						
**1.19.2 ≥ 2 years**						
Gangji et al 2011	3	13	8	11	7.1%	0.11 [0.02, 0.72]
Gangji et al 2016	3	30	6	30	8.4%	0.44 [0.10, 1.97]
Hauzeur et al 2019	12	26	6	27	9.7%	3.00 [0.91, 9.87]
Hernigou et al 2018	35	125	90	125	12.2%	0.15 [0.09, 0.26]
Hoogervorst et al 2022	15	61	15	24	10.4%	0.20 [0.07, 0.54]
Mao et al 2015	4	48	5	41	8.9%	0.65 [0.16, 2.62]
Zhao et al 2012	2	53	10	44	8.1%	0.13 [0.03, 0.65]
**Subtotal (95% CI)**		**356**		**302**	**64.8%**	**0.33 [0.14, 0.80]**
Total events	74		140			
Heterogeneity: Tau² = 1.00; Chi² = 23.97, df = 6 (P = 0.0005); I² = 75%						
Test for overall effect: Z = 2.45 (P = 0.01)						
**Total (95% CI)**		**456**		**401**	**100.0%**	**0.35 [0.17, 0.72]**
Total events	98		181			
Heterogeneity: Tau² = 1.05; Chi² = 39.98, df = 11 (P < 0.0001); I² = 72%						
Test for overall effect: Z = 2.85 (P = 0.004)						
Test for subgroup differences: Chi² = 0.01, df = 1 (P = 0.92), I² = 0%						

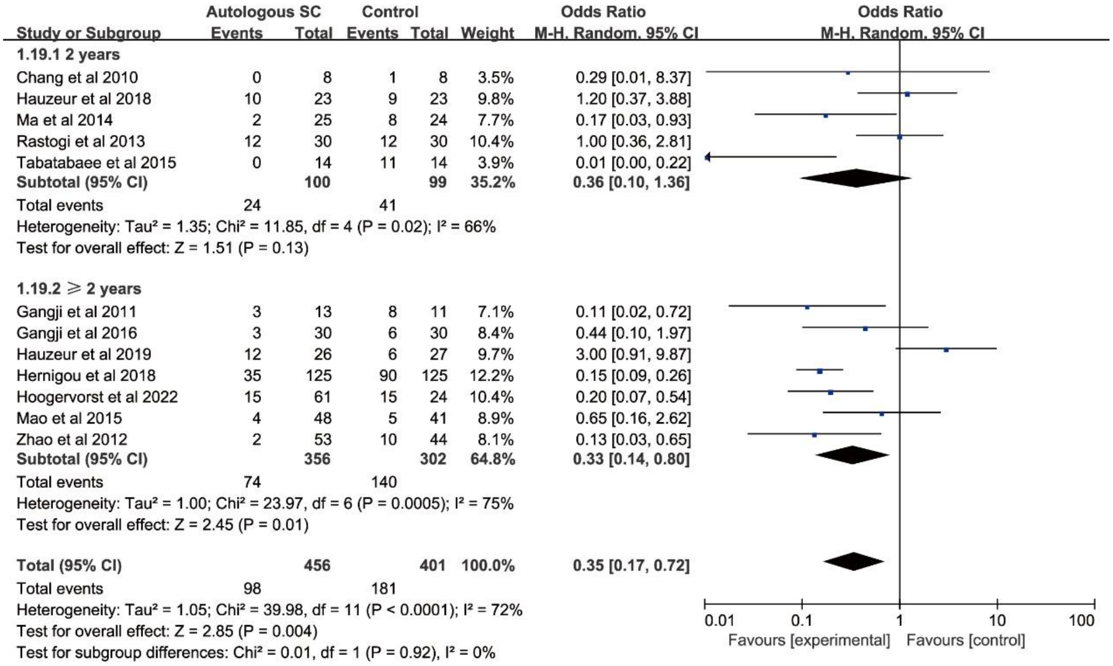

Supplementary Figure S3. Forest plot illustrating the effects of SC on femoral head collapse based on different follow-up durations.

Study or Subgroup	Autologous SC Mean	Autologous SC SD	Autologous SC Total	Control Mean	Control SD	Control Total	Weight	Std. Mean Difference IV, Fixed, 95% CI
Hauzeur et al 2018	9.9	2.6	23	9.3	2.2	23	40.3%	0.24 [-0.34, 0.83]
Hoogervorst et al 2022	23.7	14.7	61	16.4	22.4	24	59.7%	0.42 [-0.06, 0.90]
**Total (95% CI)**			**84**			**47**	**100.0%**	**0.35 [-0.02, 0.72]**
Heterogeneity: Chi² = 0.21, df = 1 (P = 0.65); I² = 0%								
Test for overall effect: Z = 1.86 (P = 0.06)								

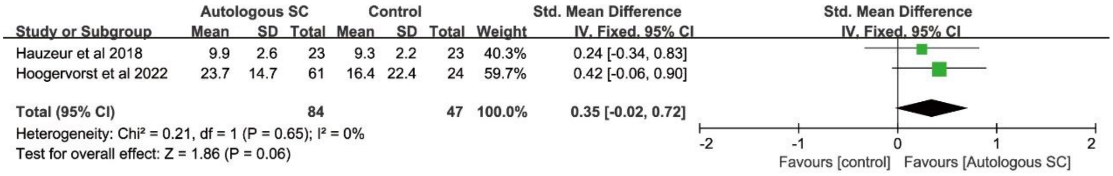

Supplementary Figure S4. Forest plot illustrating the average time for femoral head collapse.

VAS at 24 months

Study or Subgroup	Autologous SC Mean	SD	Total	Control Mean	SD	Total	Weight	Std. Mean Difference IV, Random, 95% CI
**1.21.1 2 years**								
Hauzeur et al 2018	39	5.9	23	56.1	6.4	23	12.6%	-2.73 [-3.55, -1.91]
Li et al 2021	31.8	10.5	22	26.9	12	29	13.1%	0.42 [-0.14, 0.98]
Ma et al 2014	16.92	3.66	25	26.46	2.6	24	12.6%	-2.95 [-3.77, -2.12]
Pepke et al 2016	2.26	0.99	11	2.68	0.26	14	12.7%	-0.60 [-1.41, 0.21]
Tabatabaee et al 2015	16	3.7	14	32.1	4.1	14	11.3%	-4.00 [-5.35, -2.65]
**Subtotal (95% CI)**			**95**			**104**	**62.4%**	**-1.92 [-3.56, -0.28]**
Heterogeneity: Tau² = 3.29; Chi² = 81.31, df = 4 (P < 0.00001); I² = 95%								
Test for overall effect: Z = 2.30 (P = 0.02)								
**1.21.2 ≥ 2 years**								
Gangji et al 2011	18.8	6.9	13	47.65	9.9	11	11.4%	-3.32 [-4.62, -2.01]
Hauzeur et al 2019	22	6	26	21	12	27	13.2%	0.10 [-0.44, 0.64]
Li et al 2020	2.82	0.87	21	3	1.11	20	13.1%	-0.18 [-0.79, 0.44]
**Subtotal (95% CI)**			**60**			**58**	**37.6%**	**-0.98 [-2.42, 0.46]**
Heterogeneity: Tau² = 1.43; Chi² = 22.89, df = 2 (P < 0.0001); I² = 91%								
Test for overall effect: Z = 1.34 (P = 0.18)								
**Total (95% CI)**			**155**			**162**	**100.0%**	**-1.58 [-2.67, -0.48]**
Heterogeneity: Tau² = 2.29; Chi² = 116.27, df = 7 (P < 0.00001); I² = 94%								
Test for overall effect: Z = 2.83 (P = 0.005)								
Test for subgroup differences: Chi² = 0.72, df = 1 (P = 0.40), I² = 0%								

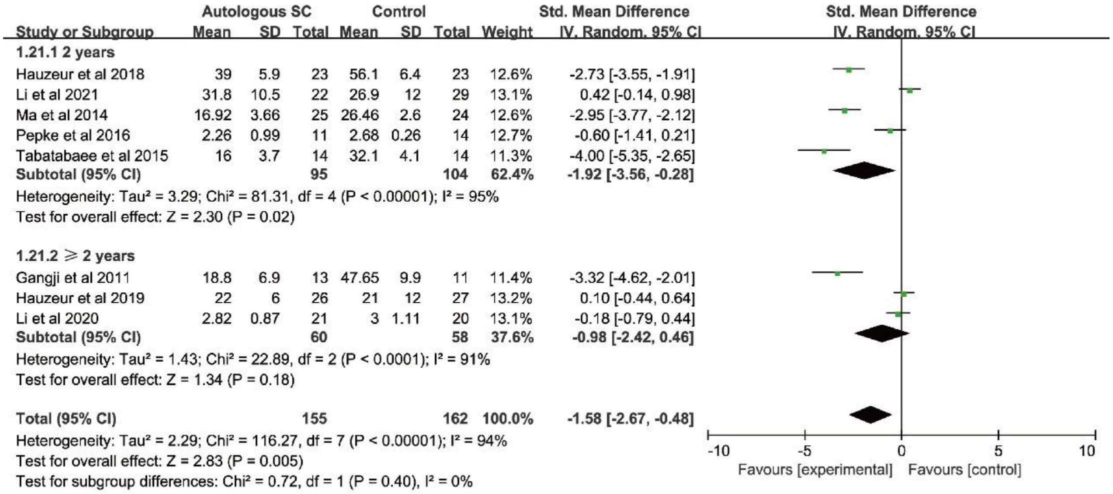

WOMAC at 24 months

Study or Subgroup	Autologous SC Mean	SD	Total	Control Mean	SD	Total	Weight	Std. Mean Difference IV, Random, 95% CI
**1.22.1 2 years**								
Jayankura et al 2023	18.6	21	25	23.8	21	29	15.2%	-0.24 [-0.78, 0.29]
Li et al 2021	52.3	6.5	22	45.9	13.9	29	15.1%	0.56 [-0.01, 1.12]
Ma et al 2014	14.81	2.99	25	22.05	3.04	24	14.5%	-2.36 [-3.11, -1.62]
Tabatabaee et al 2015	9.7	1.8	14	27.2	3.7	14	9.6%	-5.84 [-7.65, -4.03]
**Subtotal (95% CI)**			**86**			**96**	**54.4%**	**-1.77 [-3.56, 0.02]**
Heterogeneity: Tau² = 3.07; Chi² = 71.62, df = 3 (P < 0.00001); I² = 96%								
Test for overall effect: Z = 1.94 (P = 0.05)								
**1.22.2 ≥ 2 years**								
Hauzeur et al 2019	18	8	26	33	11	27	14.9%	-1.53 [-2.15, -0.91]
Hernigou et al 2018	8.6	2.3	125	12.5	2.3	125	15.8%	-1.69 [-1.98, -1.40]
Li et al 2020	17.03	6.68	21	25.11	11.41	20	14.8%	-0.85 [-1.50, -0.21]
**Subtotal (95% CI)**			**172**			**172**	**45.6%**	**-1.42 [-1.90, -0.94]**
Heterogeneity: Tau² = 0.11; Chi² = 5.43, df = 2 (P = 0.07); I² = 63%								
Test for overall effect: Z = 5.77 (P < 0.00001)								
**Total (95% CI)**			**258**			**268**	**100.0%**	**-1.48 [-2.37, -0.59]**
Heterogeneity: Tau² = 1.27; Chi² = 97.13, df = 6 (P < 0.00001); I² = 94%								
Test for overall effect: Z = 3.27 (P = 0.001)								
Test for subgroup differences: Chi² = 0.14, df = 1 (P = 0.71), I² = 0%								

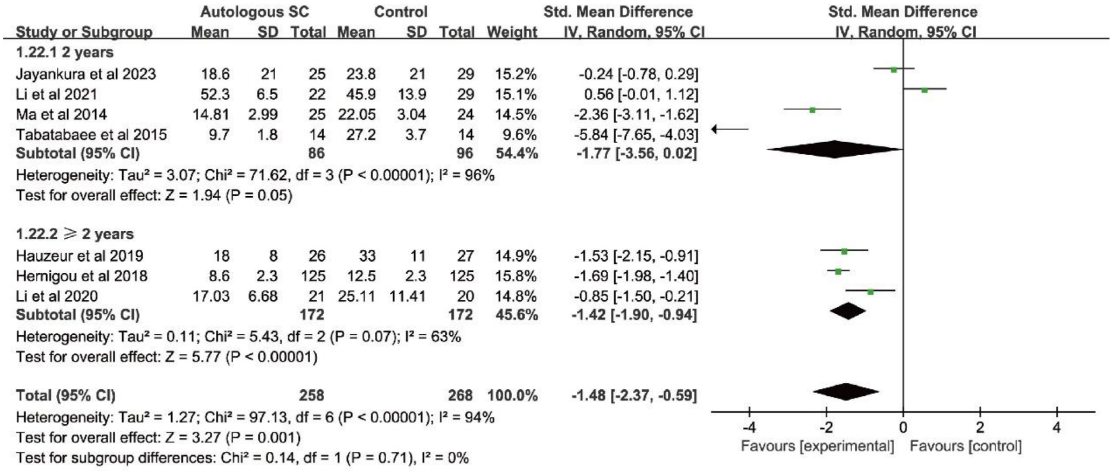

Supplementary Figure S5. Forest plot illustrating the effects of SC on VAS and WOMAC scores based on different follow-up durations.

Number of hip at stage I

Study or Subgroup	Autologous SC Events	Autologous SC Total	Preoperative Events	Preoperative Total	Weight	Odds Ratio M-H, Fixed, 95% CI
**1.10.1 Moderate dose (1-10×10^7)**						
Hauzeur et al 2018	1	23	2	23	8.5%	0.48 [0.04, 5.66]
Jayankura et al 2023	7	25	6	25	19.2%	1.23 [0.35, 4.37]
**Subtotal (95% CI)**		**48**		**48**	**27.6%**	**1.00 [0.33, 3.04]**
Total events	8		8			
Heterogeneity: Chi² = 0.45, df = 1 (P = 0.50); I² = 0%						
Test for overall effect: Z = 0.00 (P = 1.00)						
**1.10.2 High dose (>1×10^8)**						
Ma et al 2014	3	25	3	25	11.7%	1.00 [0.18, 5.51]
Mao et al 2015	11	48	8	48	27.3%	1.49 [0.54, 4.10]
Rastogi et al 2013	7	30	7	30	23.8%	1.00 [0.30, 3.31]
Tabatabaee et al 2015	4	14	3	14	9.5%	1.47 [0.26, 8.23]
**Subtotal (95% CI)**		**117**		**117**	**72.4%**	**1.25 [0.65, 2.39]**
Total events	25		21			
Heterogeneity: Chi² = 0.34, df = 3 (P = 0.95); I² = 0%						
Test for overall effect: Z = 0.66 (P = 0.51)						
**Total (95% CI)**		**165**		**165**	**100.0%**	**1.18 [0.67, 2.06]**
Total events	33		29			
Heterogeneity: Chi² = 0.89, df = 5 (P = 0.97); I² = 0%						
Test for overall effect: Z = 0.57 (P = 0.57)						
Test for subgroup differences: Chi² = 0.11, df = 1 (P = 0.74), I² = 0%						

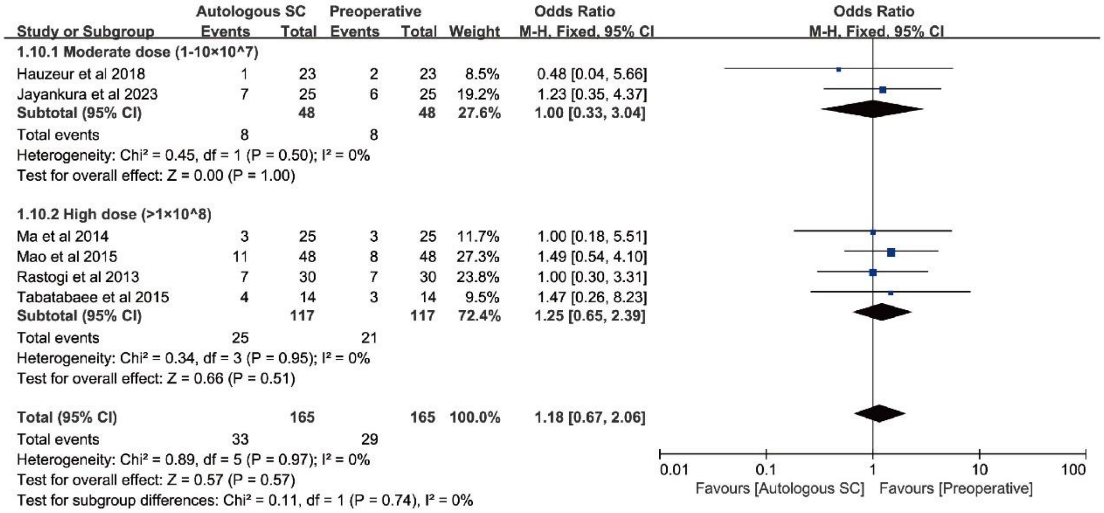

Number of hip at stage II

Study or Subgroup	Autologous SC Events	Autologous SC Total	Preoperative Events	Preoperative Total	Weight	Odds Ratio M-H, Fixed, 95% CI
**1.11.1 Moderate dose (1-10×10^7)**						
Hauzeur et al 2018	8	23	10	23	14.4%	0.69 [0.21, 2.28]
Jayankura et al 2023	12	25	19	25	21.8%	0.29 [0.09, 0.98]
**Subtotal (95% CI)**		**48**		**48**	**36.2%**	**0.45 [0.20, 1.04]**
Total events	20		29			
Heterogeneity: Chi² = 1.00, df = 1 (P = 0.32); I² = 0%						
Test for overall effect: Z = 1.86 (P = 0.06)						
**1.11.2 High dose (>1×10^8)**						
Ma et al 2014	17	25	17	25	12.0%	1.00 [0.30, 3.28]
Mao et al 2015	25	48	29	48	30.7%	0.71 [0.32, 1.60]
Rastogi et al 2013	11	30	11	30	15.4%	1.00 [0.35, 2.86]
Tabatabaee et al 2015	10	14	9	14	5.7%	1.39 [0.28, 6.84]
**Subtotal (95% CI)**		**117**		**117**	**63.8%**	**0.90 [0.53, 1.52]**
Total events	63		66			
Heterogeneity: Chi² = 0.67, df = 3 (P = 0.88); I² = 0%						
Test for overall effect: Z = 0.41 (P = 0.69)						
**Total (95% CI)**		**165**		**165**	**100.0%**	**0.73 [0.47, 1.15]**
Total events	83		95			
Heterogeneity: Chi² = 3.47, df = 5 (P = 0.63); I² = 0%						
Test for overall effect: Z = 1.36 (P = 0.17)						
Test for subgroup differences: Chi² = 1.84, df = 1 (P = 0.17), I² = 45.7%						

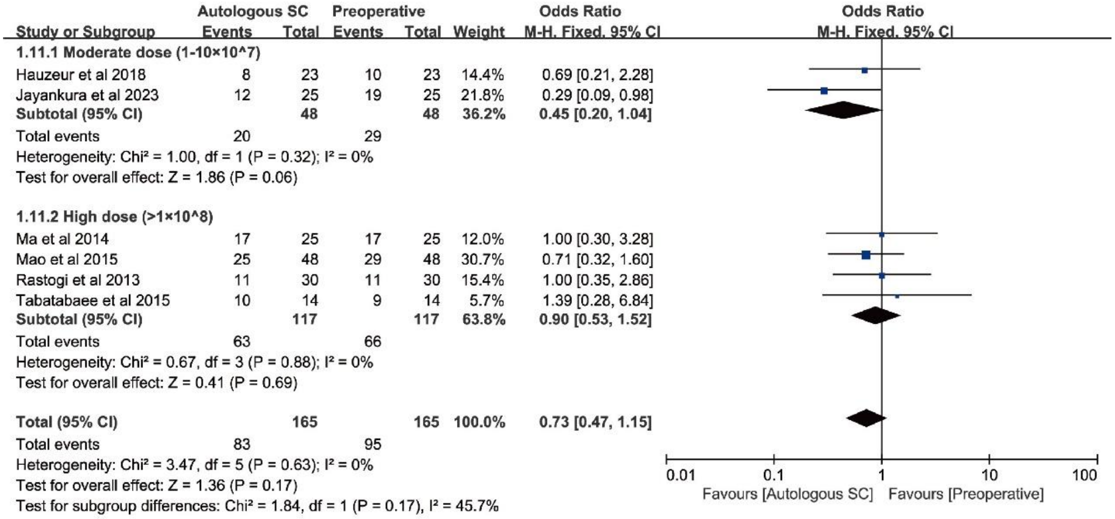

Number of hip at stage III

Study or Subgroup	Autologous SC Events	Autologous SC Total	Preoperative Events	Preoperative Total	Weight	Odds Ratio M-H, Fixed, 95% CI
**1.12.1 Moderate dose (1-10×10^7)**						
Hauzeur et al 2018	14	23	11	23	15.2%	1.70 [0.53, 5.47]
**Subtotal (95% CI)**		**23**		**23**	**15.2%**	**1.70 [0.53, 5.47]**
Total events	14		11			
Heterogeneity: Not applicable						
Test for overall effect: Z = 0.89 (P = 0.38)						
**1.12.2 High dose (>1×10^8)**						
Ma et al 2014	3	25	5	25	15.6%	0.55 [0.12, 2.58]
Mao et al 2015	10	48	11	48	30.8%	0.89 [0.34, 2.33]
Rastogi et al 2013	9	30	12	30	29.8%	0.64 [0.22, 1.87]
Tabatabaee et al 2015	0	14	2	14	8.6%	0.17 [0.01, 3.94]
**Subtotal (95% CI)**		**117**		**117**	**84.8%**	**0.67 [0.35, 1.25]**
Total events	22		30			
Heterogeneity: Chi² = 1.12, df = 3 (P = 0.77); I² = 0%						
Test for overall effect: Z = 1.27 (P = 0.21)						
**Total (95% CI)**		**140**		**140**	**100.0%**	**0.82 [0.48, 1.42]**
Total events	36		41			
Heterogeneity: Chi² = 2.92, df = 4 (P = 0.57); I² = 0%						
Test for overall effect: Z = 0.70 (P = 0.49)						
Test for subgroup differences: Chi² = 1.90, df = 1 (P = 0.17), I² = 47.5%						

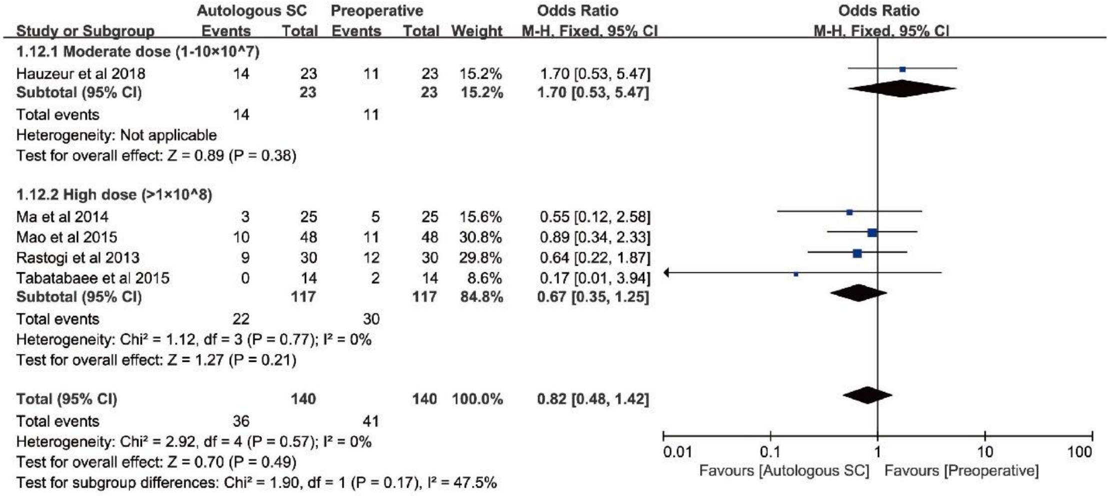

Supplementary Figure S6. Forest plot illustrating changes in ARCO stage before and after intervention with different SC dosages. (A) Number of ARCO stage I. (B) Number of ARCO stage II. (C) Number of ARCO stage III.

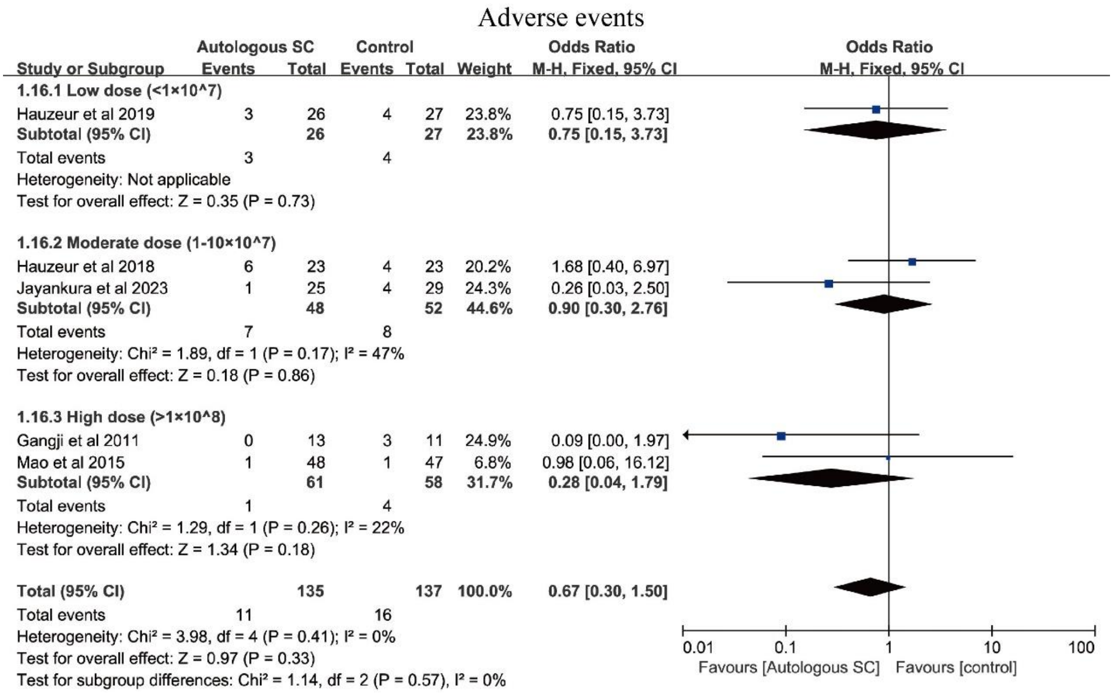

Supplementary Figure S7. Forest plot illustrating the effects of different SC dosages on the incidence of adverse events (AEs).

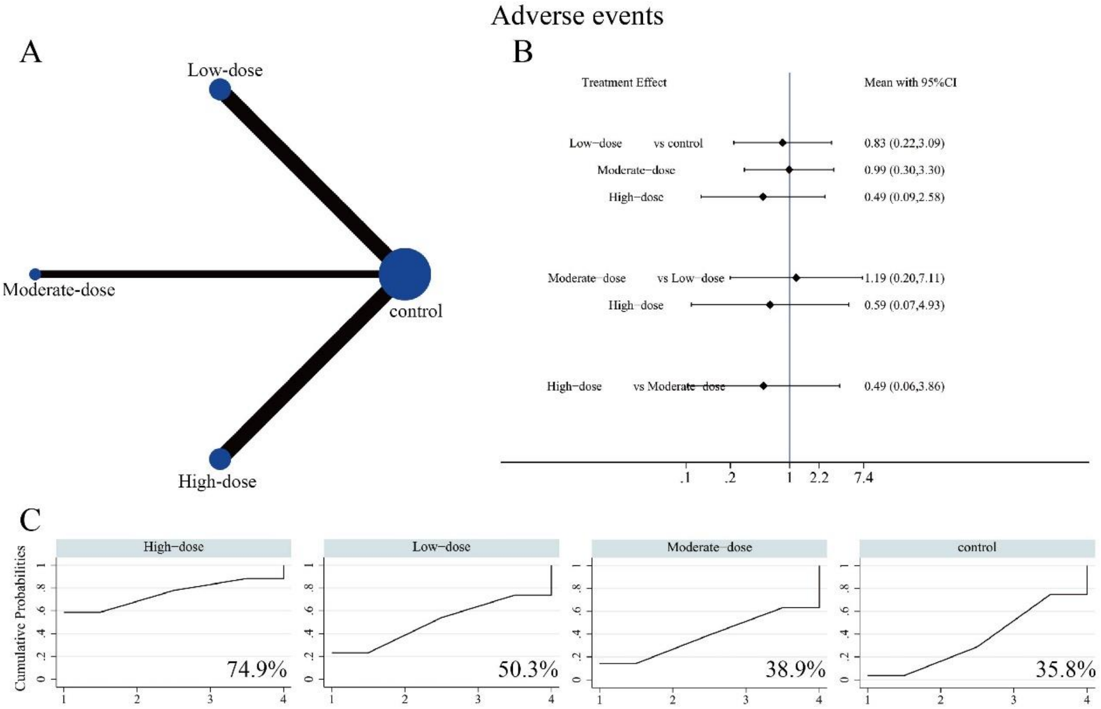

Supplementary Figure S8. Network meta-analysis of the effects of different SC dosages on adverse events (AEs). (A) Evidence network diagram for incidence of AE. (B) Forest plot presenting comparative evidence of different SC dosages on incidence of AEs. (C) SUCRA curves and surface under the cumulative ranking curve (%) for incidence of AEs after SC

intervention.

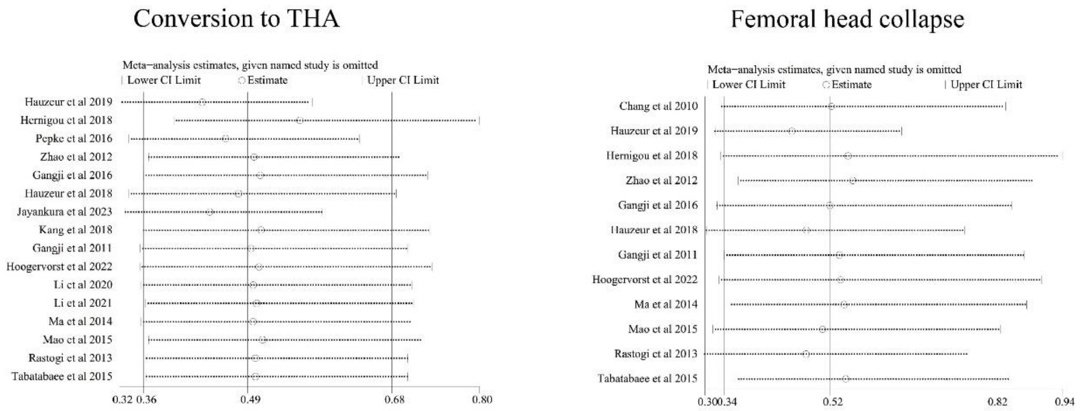

Supplementary Figure S9. Sensitivity analysis of conversion to THA and femoral head collapse in conventional meta-analysis.

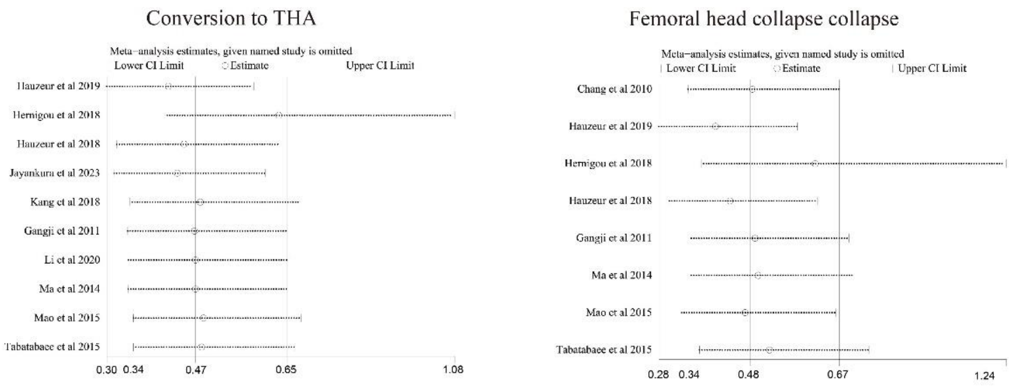

Supplementary Figure S10. Sensitivity analysis of conversion to THA and femoral head collapse in conventional meta-analysis after excluding studies with unclear dosage indicators.

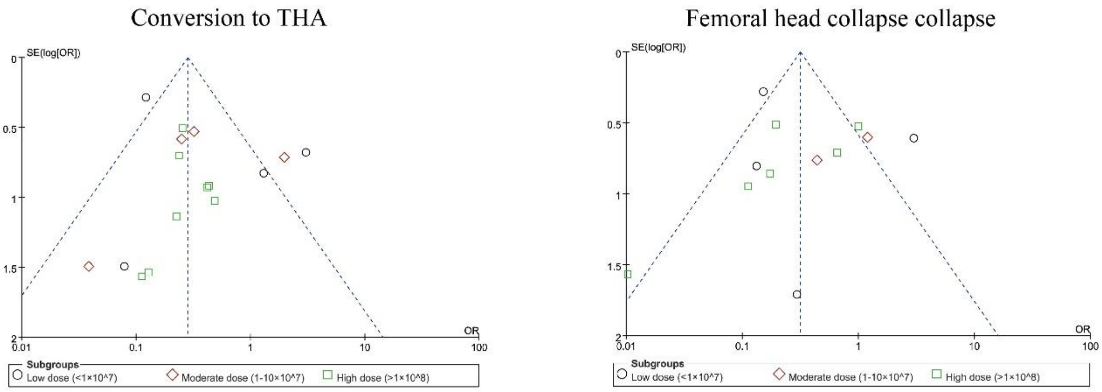

Supplementary Figure S11. Evaluation of publication bias in key indicators of conventional meta-analysis.

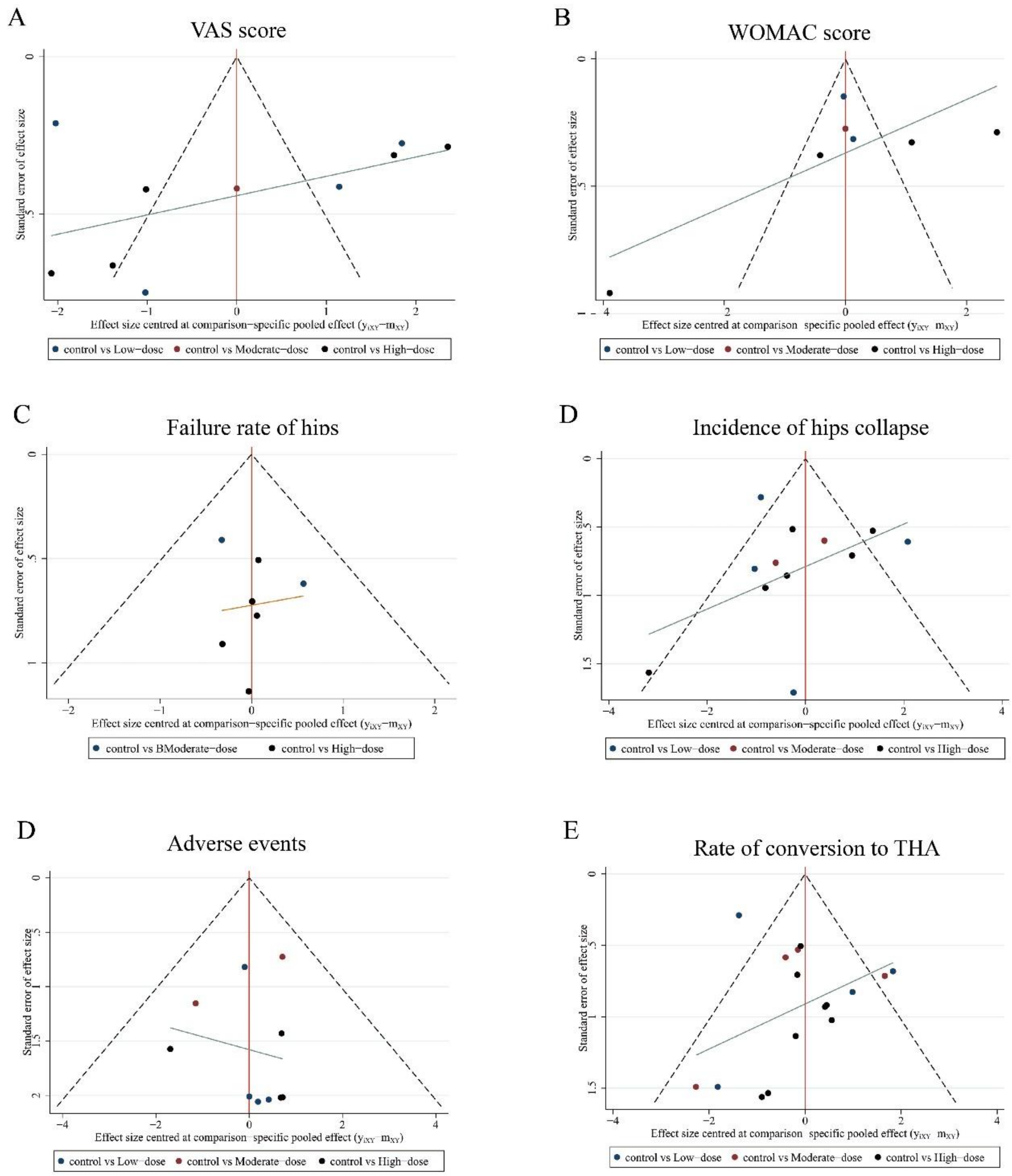

Supplementary Figure S12. Publication bias for key indicators in network meta-analyses.
